# Time to Tenure in Spanish Universities: An Event History Analysis

**DOI:** 10.1371/journal.pone.0077028

**Published:** 2013-10-08

**Authors:** Luis Sanz-Menéndez, Laura Cruz-Castro, Kenedy Alva

**Affiliations:** Institute of Public Goods and Policies (IPP), Consejo Superior de Investigaciones Científicas (CSIC), Madrid, Spain; The University of Edinburgh, United Kingdom

## Abstract

Understanding how institutional incentives and mechanisms for assigning recognition shape access to a permanent job is important. This study, based on data from questionnaire survey responses and publications of 1,257 university science, biomedical and engineering faculty in Spain, attempts to understand the timing of getting a permanent position and the relevant factors that account for this transition, in the context of dilemmas between mobility and permanence faced by organizations. Using event history analysis, the paper looks at the time to promotion and the effects of some relevant covariates associated to academic performance, social embeddedness and mobility. We find that research productivity contributes to career acceleration, but that other variables are also significantly associated to a faster transition. Factors associated to the social elements of academic life also play a role in reducing the time from PhD graduation to tenure. However, mobility significantly increases the duration of the non-tenure stage. In contrast with previous findings, the role of sex is minor. The variations in the length of time to promotion across different scientific domains is confirmed, with faster career advancement for those in the Engineering and Technological Sciences compared with academics in the Biological and Biomedical Sciences. Results show clear effects of seniority, and rewards to loyalty, in addition to some measurements of performance and quality of the university granting the PhD, as key elements speeding up career advancement. Findings suggest the existence of a system based on granting early permanent jobs to those that combine social embeddedness and team integration with some good credentials regarding past and potential future performance, rather than high levels of mobility.

## Introduction

Universities worldwide compete to improve their reputations in local and global academic markets, and attracting talent has become a key dimension in this competition [1]. The mobility of academics has also become a totem of knowledge sharing and circulation and technology transfer [2]. There is a worldwide trend for new performance-based funding models of universities [3]. As a result, increasing attention has turned to productivity and mobility as characteristics sought for academics, with policy makers and university managers emphasizing the need to select the best possible researchers [4].

The most effective ways of hiring excellent academics depend, however, among other factors, on the diverse governance structures of universities [5] and the incentive structure emerging from institutional arrangements and resource distribution. Despite public acceptance of the Mertonian normative model and the existence of evaluation and promotion regulations in many universities, little is known about the extent to which the processes of hiring and granting permanent jobs are based on productivity or other factors and to what extent mobility contributes to accelerating career advancement.

The analysis of the labor markets of academics and their careers, access to tenure, and academic promotion has a long tradition of studies centered on the USA (for a review, see [6]), where the tenure-track model exists and wages are subject to negotiation. However, the ability of organizations to develop strategies to recruit the best possible talent is conditioned by institutional structures and availability of resources; these are key factors for providing understanding and making sense of the career models that universities develop in practice, despite acceptance of the normative models of merit and mobility.

Because of national diversity, analysis of academic labor markets in Europe has been fragmented [7]. In many countries with different university governance structures, knowledge about recruitment, access to tenure and academic promotion as elements of the diverse career models is also partial. Exceptions are edited books (e.g. [8]), book series and special issues of some journals which have gathered together chapters on countries' academic career models.

Because the bulk of the literature on academic promotion refers to the institutional context of US research universities, the structural features of academic systems are rarely taken into account in the discussion of results. These features are essential, however, for understanding the different effects that the same set of factors could have. Universities operate in the context of institutionally embedded organizational dilemmas [9]. We know little about whether, in practice, access to a permanent academic position is governed by merit and universalism or by more parochial and particularistic factors; we also lack a proper understanding of how institutional incentives and mechanisms for assigning recognition shape access to a permanent job and the consequences of organizational strategies in academic careers and how universities cope with the dilemmas between permanence and turnover, loyalty and mobility, universalism and particularism, etc. The present analysis, constructed with empirical data from the Spanish case, aims to discover what factors are associated with this academic reward and its timing and how they relate to the incentive and opportunity structures and resource endowment that organizations have. To provide the international reader with some basic elements for understanding the case, Table S1 in File S1 shows some of the distinctive features of the functioning and governance of the Spanish university system that also characterize other university systems in Europe and Latin America [10-14].

The tenure model in Spain is not based on tenure-track positions [15]. In the period of interest, individuals could get a tenured position after an open tournament, based on public exams, to which all academics with a PhD could apply; formally all candidates (inside and outside the department) have the same opportunities to get the position and compete among themselves.

We are particularly interested in the role of mobility in career advancement. Some organizations respond to the dilemma between mobility and loyalty by developing strategies or incentives and creating structures of opportunities, based on rewarding commitment (and, if possible, performance). Inbreeding practices are accepted and could play a role similar to the one analyzed in the US universities many years ago [16,17]. The key to that strategic structural response to recruitment and commitment is providing early tenure. Analyzing time to promotion as part of the reward system allows us to grasp empirically some of the university system’s structural features.

In this type of system, understanding the timing of tenure and the transition to a first permanent position is essential in order to know how departments are able to commit promising candidates to the organization. This capability is a key structural feature allowing the Spanish university to compete with other institutions that could negotiate salaries and working conditions.

In this paper we do not analyze individuals who did not succeed in attaining employment permanence because the focus is not tenure versus non-tenure as a labor market outcome. In addition to the relevant argument about why it is important to study the timing for those that achieved tenure, we have to anticipate that our system is similar to some others (e.g., Canada) characterized by an association between permanence and tenure [18] in which virtually all academics that stay on at an institution eventually get tenure.

Previous studies of academic promotion have been dominated by cross-sectional designs; however, these methodologies have important deficits regarding the treatment of time [19]. In this paper, we use a longitudinal analysis and survival models to explore time to promotion and its covariates. We attempt to understand the transition to a permanent job (tenure) and its timing in Spanish universities and the relevant factors that account for it; in particular, we seek to deepen our knowledge about the role of academic performance, mobility and social embeddedness in the duration of the period from PhD graduation to tenure.

The paper is organized as follows. In section 2, we review some factors, based on previous research, relevant for our analyses. Section 3 describes the data and methods used. Section 4 is devoted to the empirical analysis and presentation of the findings. The paper ends with a discussion of the results and some conclusions.

## Analytical Framework

Access to an associate professor position, or permanent lifetime employment, is probably the key reward in university careers, especially in systems where wage differentials are marginal and mobility is low. Additionally, in the absence of a tenure-track system, the timing of promotion is important because it affects the capacity of organizations to attract and retain talent.

While in other areas of research the use of longitudinal models started quite early and is extended, such as the analysis of time to graduation [20,21], or the study of promotion systems in organizations or companies, as relevant factors for incentivizing and retaining valuable employees [22,23] little research has addressed academic promotion as it concerns the issue of timing. The seminal work by Long et al. [24] was an exception; more recently, users of longitudinal approaches have addressed the time to promotion differences between male and female academics, with a focus on gender gaps [25] and the effects of marriage and parenting [26,27], rather than on the general factors that account for promotion and its timing, which has been the case for other countries like Taiwan [28], Canada [29], and France [30].

We base our expectations about the time to tenure and its covariates on the identification of theoretically relevant factors and the findings of previous research on academic careers and access to tenure. We believe that we can group most of the thinking regarding promotion and transition to tenure around three main explanations (academic performance, social embeddedness, and mobility). Accordingly, we have included most of the relevant variables that have been used in previous research under three sets of factors. We adopt this structure for the sake of clarity but it is not meant to imply theoretical or analytical disconnection.

### 1.1. Academic performance

Research productivity, usually associated with publication performance, has been identified as a central element in academic performance; thus, in a merit-based system, it should play a central role in the advancement of careers [31,32]. Moreover, cumulative advantage career processes [33] have been clearly identified whereby we should expect early publication [34] to play a positive role in advancement in rank.

Long et al. [24] focused specifically on the length of time to promotion. Variation in timing of promotion was a key component of the dependent variable, i.e., whether or not the scientist was promoted, and, if promoted, how many years elapsed before the event. Overall, they found that promotion to an associate professorship was influenced by the number of articles published while at the rank of assistant professor. Interestingly, their results showed that articles published prior to obtaining the position of assistant professor (first postdoctoral position) had no significant effects on the rate of promotion. Long et al. [24] also found a lower expected probability of promotion for women; in fact, the positive effect of publications was found to benefit only a minority of extremely productive women. The problem for generalization of the results was that their sample was field specific, composed only of biochemists.

Other classical variables used in predicting future performance of candidates have been the reputation of the universities granting the doctoral degree and indicators of fast educational attainment, such as short times to obtaining a doctoral degree [35]. Those variables could be considered relevant in contexts of incomplete information and uncertainty about future potential and performance of the candidates, as in early professional career advancement; in these contexts, departments use cognitive devices [36] in selection and promotion processes, which could help in making decisions.

### 1.2. Social embeddedness

The idea of the strength of weak ties and the concept of social embeddedness [37] express the notion that social actors exist within relational, institutional, and cultural contexts; thus, the results of promotion processes could also be shaped by this embeddedness. Research and science are not individual but collective enterprises, and to advance in their careers, researchers also need some level of established social capital [38].

The set of factors related to social embeddedness of researchers (including elements of social capital) consists of variables linked to research and organizational contexts and may sometimes be shaped by “particularistic” processes associated with loyal integration into the local environment. To be socially integrated means to be part of research groups (especially important in the experimental sciences), which are the place of the collective enterprise of the research activity. Classical and recent studies have emphasized the relevance of research teams in science [39,40], networking and collaboration are part of researchers’ regular activities [41] and have a positive impact on promotion [42]; it has also been argued that involvement of researchers in teams is relevant for performance [43] and promotion.

A specific type of social capital in academic life is acquired in the context of the relationship with the PhD supervisor if he/she becomes the source of mentorship [44]; research collaboration with the PhD supervisor after the PhD could play a positive role in the academic career, increase the visibility of the work of the researcher, and send signals to colleagues that he or she is integrated in the community [45].

In addition, the involvement of faculty in management tasks and academic structures of universities, what has been called “institutional service”, could increase social interaction at an institutional level and improve the perceived contribution of candidates to university activities, other than academic performance. It could be claimed, however, that task diversification might, especially early in careers, hinder publications [46].

Finally, another indicator of social embeddedness, overlapping with mobility, is inbreeding (lack of mobility between the PhD-granting university and the university granting tenure [16,17]); staying in the same university for the whole career could have the effect of reinforcing the relations with the local environment, and previous studies for Spanish researchers found that inbred faculty were at a relative advantage of getting early tenure compared to the non-inbred, although the relationship was barely statistically significant [47]. These factors, not necessarily correlated with productivity and performance, are likely to increase the social integration of candidates into the local and organizational environment and create a context in which social familiarity and proximity [48] could emerge as particularistic criteria; there is also a reasonable expectation that being actively networked and integrated in the organizational context in which the promotion occurs will ease the transition and reduce time to tenure.

### 1.3. Mobility

Mobility is supposed to contribute to knowledge circulation and, in the context of well-functioning academic markets, it could play a positive role in the advancement of careers through competition among employers. In principle, one could expect some academic systems to promote mobility strongly while other systems and universities incentivize retention of people and reward loyalty [17]. Geographical mobility has long been claimed to be an important factor in promotion [49,50] and even to have a key role in gender differences [51], but there are several different forms of mobility to be considered.

A specific valuable form of mobility is having earned the PhD abroad. In many countries, the reputation of having a foreign degree could be considered a relevant career factor. Tien [28] found for Taiwan that although foreign-trained faculty may enjoy a certain prestige in society, they were not advantaged over their domestically trained counterparts, so that controlling for other variables, their chances of reaching either associate or full professorship were the same. She found that the number of publications was a good predictor of the odds for promotion, but whether the system was truly universalistic remained an open question because female and younger faculty were disadvantaged when it came to seeking promotion.

We acknowledge that mobility may affect productivity since it exposes scholars to new environments that affect their activities and more productive individuals may also be more mobile [52]. Internationally mobile researchers (especially postdoctoral) have access to international networks and socialization and to opportunities of increasing their publications [53]. It is claimed that there is an optimal length of stays abroad [54] that maximizes returns in terms of productivity and advancement of careers; however, this type of mobility could also have negative career effects [55] because these mobile researchers could face a more difficult integration in local environments. In this vein, previous findings evaluating the impact of mobility on early career in Spain revealed that it mainly delays career advancement [47]; when it made a positive contribution, it was in the form of sponsored and short-term mobility [56]. A further form of mobility, moving into the non-academic labor market or into firms, could produce the effect of delaying a career in the academic world, especially in the early stages of careers, and could have negative effects on productivity in the short term [57].

An additional dimension to consider, overlapping the social integration dimension, is mobility across research groups and teams; this mobility has been identified as an essential way of promoting interdisciplinarity [58]. If contextualized in terms of the local and organizational environments that condition research activities, though, it could weaken social embeddedness and delay career advancement, at least in the early phases [43].

While the impact of mobility in academic systems where open job markets exist has usually been related to improvements in academic careers, more success and more productivity, we believe that the expected effect of mobility is likely to be conditioned by the general incentive structure of the academic system. Previous research in Spain already found that different forms of mobility affected early tenure negatively [47].

In Table 1 we summarize the expected effects of the factors and variables reviewed based on the understanding of the institutional context, resources, incentives, and opportunity structures emerging from the Spanish system.

**Table 1:** Expected effects on time to tenure of the covariates.

**Factor**	**Sub-factors**	**Variables (measured)**	**Effect on duration**
**Academic performance**	Postdoctoral Productivity	Postdoctoral Publications by year	-
	Early productivity	Early publications	-
	Reputation of university granting PhD	Research orientation of the university granting the PhD	-
	Time to PhD degree	Time to PhD degree (years)	+
**Social embeddedness**	Membership of research team	Involvement in research groups	-
	Collaboration with PhD supervisor	Collaboration with PhD supervisor	-
	Institutional service	University service	-
	Inbreeding	Inbred status	-
**Mobility**	PhD abroad	Place of the PhD (foreign)	+
	International mobility	Postdoctoral international mobility	+
	Intergroup mobility	Mobility across research groups	+
	Intersectoral mobility	Mobility outside academia	+

(+) Increases time to tenure; (-) reduces time to tenure.

### 1.4. Control variables

Some socio-demographic factors (age and sex), field of research, demand level, and reputation of universities granting tenure have been reported to be relevant. Among individual attributes, sex and age have been identified in the literature as important. Most of the literature has found relevant and significant effects against women in terms of promotion [24,59], but more recently, the effects of sex on promotion show a downward trend [60].

In addition, age and age at PhD (we take the latter variable because most previous studies confirm it as the start date of the academic career) have been reported to be important variables to account for productivity [61] and for advancement in careers [29]. Although sometimes the literature has presented promotion as a process shaped by seniority and age [62], in aggregated terms, the results are somewhat contradictory: the majority of work finds that the younger the age at PhD graduation, the faster the career advancement, despite some seniority effects reported.

Although many classical studies have analyzed single disciplines, the functioning patterns of labor markets and promotion among diverse scientific and technological fields are significantly different [63]. Sauermann and Stephan [64] point out the need not to overlook heterogeneity across fields within the academic sector. Dietz et al. [65] found evidence of much higher probabilities of tenure for engineers in comparison with biomedical academics. In principle, departments in areas with higher alternative market opportunities and economic gains (engineering and technology) will shorten the period for promotion as a strategy to attract talent. The expectation is that advancement of academic careers will be faster in areas with greater outside employment opportunities and a lower supply of PhDs.

Universities and departments with a higher reputation usually take more time to grant tenure than non-research-intensive universities [62]; some literature even finds evidence of strategic tradeoffs at the level of departments between salary offers and tenure expectations in particular fields [66].

Finally, an important variable that is missing in most previous studies refers to the differential demand of academics by universities and the evolution of new tenure positions. Recent evidence has suggested effects of the labor market situation on the quality and productivity of the selected candidates [67,68].

In addition to the set of factors that we have included in our analysis and the control variables, other variables have also been considered as relevant in the literature of career and promotions. We have not included them either because of the focus of the paper or because they were unavailable; these factors are wages, teaching load, quality of publications, family conditions (marriage or dependents), or interdisciplinarity, among others.

Pay and compensation [69] has been central in the analysis of academic labor markets, however, without individual salary differentiation and wages regulated by law, the variable was not very relevant for the current analysis. Teaching load [70], which appears to be important in influencing productivity, is not directly relevant in the process of tournaments, which are based on public exams.

We have not included measures of impact associated with citations; the reason is twofold: first, to avoid methodological problems of making comparisons across disciplines and research areas that have diverse citation models [71], citation uses [72], citation context [73] or citations delays [74]; considering those factors associated to citations will move us away from the identification of the contribution of performance to time to tenure and into a bibliometric debate; second, we build on the findings of Long, Allison and colleagues highlighting that quantity of publications is more important than quality in predicting access to first tenure position and prestige does not have a strong influence on research productivity [24,75].

Unfortunately, family conditions data were not collected in the questionnaire. We should note, however that sex is only a control variable and we found no significant differences in time to promotion between men and women. Although some argue that differences in salaries by gender were the product of the differences in timing and time to promotion and differences in the treatment of women with respect to having children [76], recent work [25] has found that family variables had no significant negative effects on the career of women, for whom marriage did not slow transition to tenure-track status, and parenting had no negative influence on tenure promotion once in tenure track. These results diverged, however, from those of [26], who found that marital status and young children accounted for the gender differences in obtaining tenure-track positions.

Regarding the influence of the degree of interdisciplinarity and specialization in career advancement in sociology it has been reported that specialization decreases the chances of promotion [77]; however, we are examining science, biomedicine, and engineering faculty working in many disciplines and boundary areas; the practical measurement of interdisciplinarity and the way we collected the publications data (without taking into account the area of classification of the journal) made its consideration untenable.

## Data and Methods

### 2.1. Data and variables

Previous research on academic careers and promotion has suffered from some important deficits, methodological problems, and limitations that call into question the generalization of results. Single-year studies and cross-sectional datasets, insufficient sample sizes, studies referred to a single scientific field or research organization, validity problems of self-reported productivity variables or, in many gender studies, a lack of productivity data, have been mentioned as factors weakening the findings [6,18,78]. We tried to cope with those weaknesses and, because the focus of our analysis is timing and time to promotion, the data collection was designed in a longitudinal way.

The universe of reference was the faculty of all scientific fields who obtained their first permanent tenure position between 1997 and 2001 at public universities, which represents most of the Spanish higher education sector. In Spain, public universities represent almost 92% of the student market and more than 95% of research activity at higher education institutions. University professors who earn a permanent position are granted “civil servant” status; 7,637 individuals obtained their first permanent position in all research domains at Spanish public universities during the years of reference (3,804 in science, biomedical, and engineering faculty). To yield valid results by scientific field (5) and size of the institution (5), a representative sample of 5,306 individuals was selected from the database (Register of Civil Servants), including faculty who received tenure at 37 universities.

Individuals were surveyed using a national mail survey conducted in 2005. A structured self-administered questionnaire addressing research and professional trajectories, including more than 40 questions, was used to construct the different individual and career variables. This project was carried out in accordance with the 1976 Declaration of Helsinki and the Código de Prácticas Científicas of the Consejo Superior de Investigaciones Científicas and the Ministerio de Ciencia e Innovación (BOE, 21 de diciembre de 2010). As a Spanish public organisation, the Consejo Superior de Investigaciones Científicas (our institution) is obliged to conduct all research in compliance with Ley Orgánica 15/1999, de 13 de diciembre, de *Protección de Datos de Carácter Personal* (Personal Data Protection Act). The legislation can be viewed at http://www.boe.es/boe/dias/1999/12/14/pdfs/A43088-43099.pdf. All respondents were informed that no personal data would be provided in a non-aggregated way, and they were asked for their consent for the distribution and publication of this information in an anonymized and aggregated way. There was no obligation to return the survey. A copy of the survey, including the information provided to the respondent, is included in the supporting information (Questionnaire in File S2).

We obtained a total of 2,588 valid questionnaires (50% response rate) with a sample error of 1.58% (at 95% and p=q) and 4.5% for the representative sub-samples. The examination of non response focused on the detection and estimation of the extent of non response bias with techniques under the label of archival and benchmarking analysis [79], comparing respondents and non-respondents on the variables used for stratification. We controlled for the two attributes used for stratification of the sample (research area and size of university). The valid questionnaires of our sample matched the distribution of the sub-populations by area of knowledge and size, after some recalling letters on the categories with lower response rates.

To complement the information gleaned from the questionnaires and considering that scientific publications in peer-reviewed journals are generally accepted as one of the most important elements for career advancement [30], we constructed a database of individual pre-tenure publication records in journals between 1990 and 2004 included in the Science Citation Index Expanded (SCI) from Thomson–Reuters, by matching the names of the individuals in our survey. In the questionnaires respondents were asked to identify a few personal publications, the regular way of signing academic papers and offered the opportunity to submit a full CV. The disambiguation process was made manually, based on the information supplied by the respondents, including the different affiliations they had and many CVs received. We included all types of publications that match the names that authors used on their papers, and we used whole counting.

Using publication as a single indication of research productivity was a decision taken acknowledging that a) we are individualizing a collective product in a single author b) teams are the standard way of functioning in research, and sometimes the junior researcher is benefiting from the group in which authorship is also an indication of scientific status [80]. However it is important to note that we have controlled for research group membership as a relevant covariate in our analysis. Of course, attributing the paper to a single author (in early career phases) could have the effect of also measuring the dimension of social embeddedness or scientific social integration [81]. We did not use self-reported publications because of the lack of reliability in reporting quantitative data of this kind.

For the present analysis, to guarantee the comparability of the measurement of scientific output (publications), we excluded individuals from social sciences and humanities, because only a limited number of researchers in those fields (less than 15% in our original sample) had any of their publications included in these databases, confirming the different publication patterns of the majority of academics in social sciences and humanities and the difficulties of analyzing scientific performance in these fields based only on international papers. Thus, our analysis covers three scientific fields, defined according the OECD Fields of Science classification: Biological and Medical Sciences, Exact and Natural Sciences, and Engineering and Technological Sciences.

We collected data with reference to the moment in which the individuals earned tenure, rather than when they earned the PhD. We are aware of the risk that this procedure involves as regards a possible bias in the absolute level of the median time elapsed to tenure [82]. Because we are interested in the differences in elapsed time to tenure among individuals getting tenure between 1997 and 2001, just before legal reform, and because the distribution is relatively stable over the years, we believe that our management of the data is reasonable. Additionally, to control for the effect of the possible biased selection on the median duration that may result from taking the tenure year as the reference in the construction of the data, we have replicated the models with adjustments in the data sets (following the practice of [20]), removing the extreme outliers. We have filtered the individuals who received a PhD before 1985; with this exercise, the sample size was reduced, but the results were quite similar.

This option is coherent with our choice of focusing our research on timing of those getting a permanent job in the context of retention strategies. As an additional control, we organized our sample by cohorts, following Kamiski and Geisler [60], and we selected the central cohorts as an additional way of reducing the potential bias of the estimates.

Our data set is complete and has no right censorship because all of the units obtained promotion. The final size of our valid dataset for this analysis was 1,257 science, biomedical, and engineering faculty (32% of them women, representing the same percentage as in the universe of reference), with a total of 7,256 years at risk, the majority of whom are in their mid-careers (mostly in their early forties, with an average age in 2005 of 42 years, a median and mode of 41, and an average of 5.8 years after tenure). The anonymized dataset is available upon request. Table S2 in File S1 presents the variables that were operationalized in our models, with the explanations, sources of data, and units of reference (observations).

Table 2 and Table 3 give the descriptive statistics of the quantitative and categorical dependent variables. Table 4 and Table 5 show the descriptive statistics of the different categories of the variables in relation to the dependent variable; for the quantitative variables, the estimated length of time to tenure is calculated with reference to the position with respect to the mean value. Table S3 in File S1 presents the correlations among variables; we observe that coefficients are generally low; exceptions are age at PhD with Time to PhD, and early publications with postdoctoral publications (but there is not collinearity among them).

**Table 2:** Descriptive statistics of the quantitative variables.

**Quantitative variables**	**Mean**	**Median**	**SD**
Time to tenure (years)	5.8	5	3.4
Postdoctoral Publications by year	2.9	1.0	5.90
Early publications	1.5	0.2	4.64
Time to PhD degree (years)	6.6	6	3.17
Age at PhD (years)	30.5	29	3.86

N valid = 1,257 cases.

**Table 3:** Descriptive statistics of the categorical variables.

**Categorical variables**		**Mean**	
**Research orientation of the university granting the PhD**	High (reference)	0.29	
	Medium	0.37	
	Low	0.34	
**Involvement in research groups (yes)**		0.94	
**Collaboration with PhD supervisor (yes)**		0.81	
**University service (yes)**		0.55	
**Inbred status (yes)**		0.71	
**Place of the PhD (foreign)**		0.02	
**Postdoctoral international mobility:**	No (Reference)	0.47	
	Yes, short stay (<6 months)	0.24	
	Yes, long stay	0.29	
**Mobility across research groups (yes)**		0.27	
**Mobility outside academia (yes)**		0.03	
**Sex (men)**		0.68	
**Research field:**	Biology and Biomedical Sciences (reference)	0.29	
	Exact and Natural Sciences	0.37
	Engineering and Technological Sciences	0.34
**Research orientation of the university granting tenure**	High (reference)	0.31
	Medium	0.33
	Low	0.36
**University demand level:**	Low growth (reference)	0.37
	Medium growth	0.30
	High growth	0.33

N valid = 1,257 cases.

**Table 4:** Quantitative variables and time to tenure.

		**Duration (in years)**		
**Quantitative variables**		**N**	**Mean**	**SD**
Postdoctoral publications by year (ln)	<mean	697	5.57	3.52
	≥ mean	560	6.03	3.22
Early publications (ln)	<mean	851	6.27	3.72
	≥mean	406	4.74	2.28
Time to PhD	<mean	801	6.08	3.32
	≥mean	456	5.23	3.48
Age at PhD	<mean	782	6.27	3.35
	≥mean	475	4.95	3.32

**Table 5:** Categorical variables and time to tenure.

		**Duration (in years)**		
**Categorical variables**		**N**	**Mean**	**SD**
Research orientation of the university granting the PhD	High	355	4.87	3.27
	Medium	470	6.63	3.53
	Low	432	5.58	3.14
Involvement in research groups	No	78	7.18	3.79
	Yes	1179	5.68	3.35
Collaboration with PhD supervisor	No	244	6.64	3.75
	Yes	1013	5.56	3.28
University service	No	569	6.18	3.41
	Yes	688	5.43	3.36
Inbred status	No	364	6.29	3.52
	Yes	893	5.56	3.33
Place of the PhD	Spain	1235	5.76	3.41
	Foreign	22	6.50	2.97
Postdoctoral international mobility	No	600	4.96	3.33
	Yes, short stay (< 6 months)	296	5.83	3.44
	Yes, long stay	361	7.08	3.05
Mobility across research groups	No	917	5.48	3.39
	Yes	340	6.55	3.32
Mobility outside academia	No	1217	5.67	3.30
	Yes	40	8.90	4.74
Sex	Women	398	6.44	3.18
	Men	859	5.46	3.45
Research field	Biology and Biomedical Sciences	371	7.64	3.34
	Exact and Natural Sciences	463	6.02	3.00
	Engineering and Technological Sciences	423	3.87	2.82
Research orientation of the university granting tenure	High	388	5.63	3.49
	Medium	418	6.11	3.52
	Low	451	5.58	3.18
University demand level	Low growth	461	6.66	3.43
	Medium growth	375	5.64	3.48
	High growth	421	4.93	3.05

### 2.2. Modeling time to tenure

Our key variable of interest measures the period (time to tenure) that an academic spends as a PhD before gaining tenure, but we are also interested in the relationships between the observed duration and some covariates of theoretical interest previously identified in the literature.

Most previous research on rank advancement, access to tenure, and promotion has used cross-sectional approaches and focused on the probabilities of obtaining a rank. However, this type of approach is not appropriate for addressing the problem of timing of an event that occurs at different times for different individuals.

Our units of analysis (individual researchers) are in a state (PhDs without tenure) that allows them to take part in the permanent position competitions (tournaments) at the universities. Units are observed over time from the year of earning the PhD and, at any given time, each unit is “at risk” of experiencing the event (getting a permanent job). We monitor their survival time in the duration process, modeled as probability of survival, hazard rate or rate of transition. Then we use event history analysis to model both the length of time spent in the initial state and the transition to a subsequent state (that is, the event).

The simplest approach to this kind of data is to use a regression model to predict the timing of transitions and discrete time or event history models, which focus on the probability of a transition from one state to another. Because inspection of the data reveals a non-linear relationship with time, a standard ordinary least squares regression is not appropriate.

The dominant modeling used for the analysis of risk of promotion has been proportional hazard models (in addition to [24], see for example [57,65,76,83]), also known as Cox proportional hazard, or semi-parametric, models; these models require the assumption that the effects of predictor variables do not change over time, so each one can be represented by a single coefficient. This type of analysis is generally recommended [19] unless timing dependency is substantively meaningful (as opposed to a nuisance), which is our case. Additionally, previous analysis has been built based on discrete event modeling and not on continuous event analysis [77]. Inspection of our data showed that the effect of different variables violates this assumption, and the probability of being promoted changes with time.

The set of accelerated failure time (AFT) models does not require these assumptions because they are parametric models that vary in form according to the observed distribution of failure times. AFT models look just like conventional regression where the time until promotion is taken as the dependent variable and the regression coefficients as estimates of the effects of the predictors on the number of years to promotion. The principal advantage of parametric duration models is their ability to provide parameter estimates while simultaneously producing relatively simple and easy-to-interpret characterization of the baseline hazard rate. Event history analysis (EHA) through the use of parametric models is better suited to dealing with non-linear processes and to estimating the function of transition to tenure; at the same time most EHA methods, carefully used, allow us to incorporate information on time-varying covariates (TVC) [19].

We have also controlled for unobserved heterogeneity, an important issue in case there are theoretically relevant variables not included in the model. The practical way to deal with unobserved heterogeneity in transition rate models is to incorporate an “error term” into the model specification [24]. In our case, we have estimated the log-logistic model with a Gamma Mixture (the way suggested to deal with the issues [84,85]), and we have found that the variance of the gamma distribution is not significant, i.e., not greater than zero. This proves that there is insufficient evidence for the existence of an unobserved heterogeneity in our model, and thus we have not introduced the error term in the specification.

## Results

We have used econometric modeling for estimating the determinants of time to tenure. To find the best fit, we test different functional forms of the relationship of time with the risk of transition: four parametric models (Exponential, Weibull, Gompertz, and Log-Logistic). To compare the adjustment, we also used one semi-parametric model (Cox). The specific features, properties, density, survival, and expected duration functions can be found in the literature [19,84]; in Annex in File S3, we present the specifications of the model.

**Figure 1:**
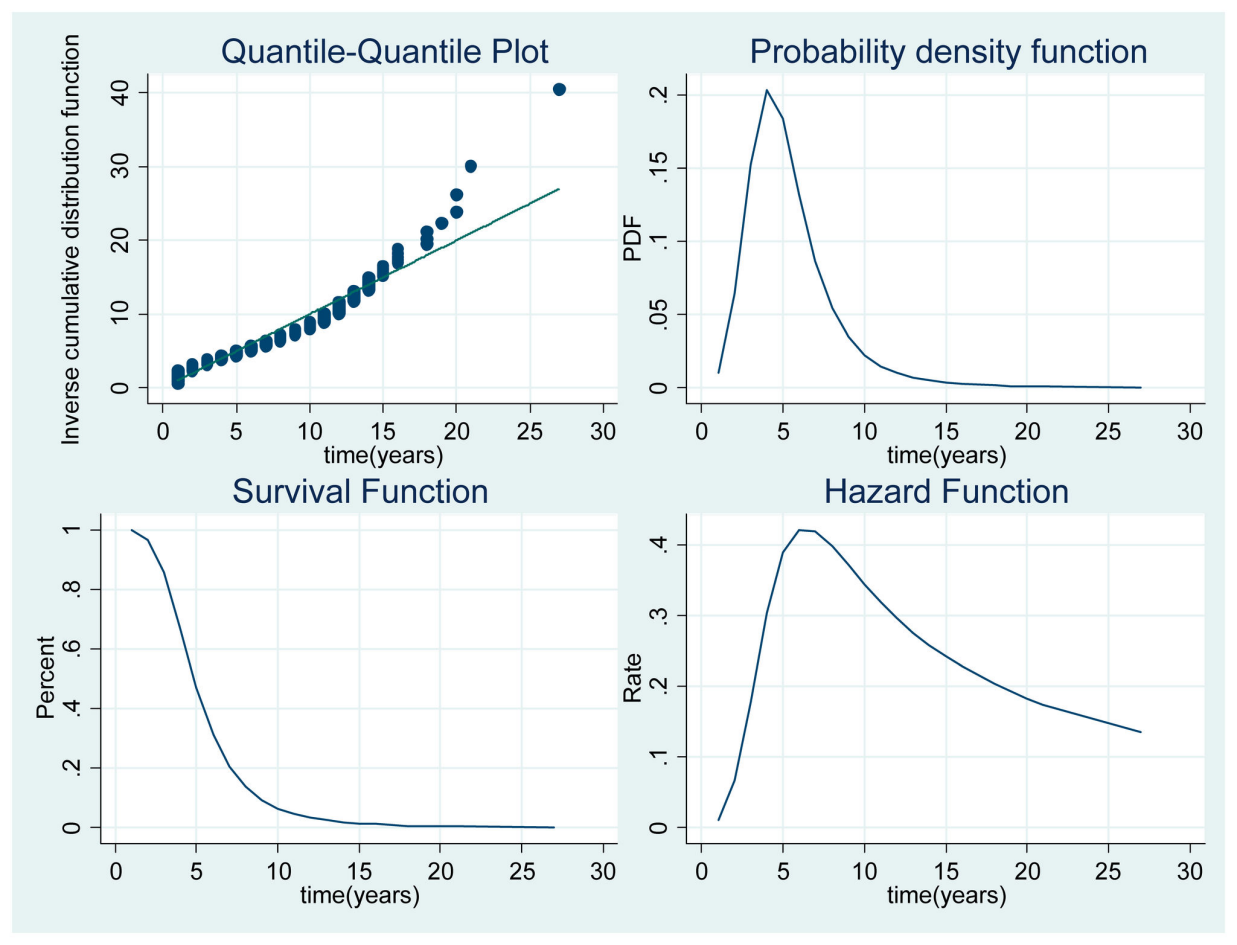
Parametric survival function, probability density, and hazard functions.

In Figure 1, we present the parametric survival functions, density functions, and hazard functions. Because the shape parameter in the transition rate is greater than one, the graph is bell shaped, explaining why the log-logistic regression model fits the data. Also a visual inspection of the data suggests that a log-logistic function (inverted U shape in the hazard function) provides the best fit to the data. By the shape of the plot, it looks like timing of access to a permanent job in Spain could be described by a log-logistic model. The Quantile-Quantile plot presented confirms the results.

The probability density function shows that the exit rate is higher in the first 4 years after the PhD. In the first year, departure rates are somewhat lower whereas in the next 4 years tenure rates are high. The survival function shows a rather steeper decline in faculty at early times and a more moderate descent at later times. Clearly, one group has fast access to tenure (early tenure) while others leave for permanent employment at a lower rate. The hazard function tells a similar story. It has relevant, non-linear changes over time and represents the rate of attrition at a given point in a faculty career, peaking at about 6 years and then dropping. The curve is like a diffusion curve where the rate of transition increases monotonically at the beginning and then starts to drop, producing an inverted U shape.

In Table S4 in File S1, we present a comparison of results for the various specifications of the duration models, including time-varying covariates, and the log-likelihood, LR Chi-square, and Akaike statistics. The log-logistic model has the lowest value (Akaike = 1847.7), identifying it as the most parsimonious model [19] and therefore as the best adjusted. The LR test (697.61) is statistically significant (p < 0.05). On the basis of the comparative results, we confirm that time to tenure seems to follow a log-logistic distribution as the first descriptive analysis suggested.

Estimates of the duration of the log-logistic model for time to tenure, for the complete sample and for each of the 3 research fields, are presented in Table 6. To facilitate interpretation, the coefficients are reported in the left column as time ratios; a value higher than 1 means that the variable produces the effect of increasing the duration while a time ratio value below 1 means that the variable contributes to the acceleration of the transition. In this table, in addition to reporting the coefficients as time ratios, we also report in the right column the marginal effect or differences in the expected duration (in years) of each covariate. These differences in the expected duration are calculated as the sample means of the regression variables (equation 4 of Annex in File S3); they indicate the expected change in elapsed time to promotion (in years) if the explanatory variable increases by one standard deviation (σ, sigma) for quantitative variables or changes to 1 for binary variables, all other variables held constant. Most of the coefficients of the variables are significant at the level of p < 0.05 or better. To get a better fit of the logistic regression model, we have tested the interaction effects between different pairs of variables to determine their inclusion in the models. The only significant interaction was Research field × Post doctoral publications where the coefficients are positive, i.e., if the interaction of both variables increases, it takes longer to get tenure; the area in which publications are less important for advancement and where the negative effect is biggest is Engineering and Technologies.

**Table 6:** Log-logistic survival model and expected length of time to tenure (general and by Research field).

		**General**			**Biology and Biomedical Sciences**			**Exact and Natural Sciences**			**Engineering and Technological Sciences**		
**Variables**		**Time ratio**	**P>|z|**	**Diff (years).**	**Time ratio**	**P>|z|**	**Diff (years).**	**Time ratio**	**P>|z|**	**Diff (years).**	**Time ratio**	**P>|z|**	**Diff (years).**
Early publications (ln)		0.783	0.000	-0.86	0.752	0.000	-1.16	0.741	0.000	-1.02	0.889	0.067	-0.32
Postdoctoral publications by-year (ln)		0.985	0.604	-0.07	1.011	0.707	0.08	1.099	0.002	0.49	1.055	0.410	0.18
Research orientation of the university granting the PhD (reference: High)	Medium	1.216	0.000	0.71	1.218	0.010	0.90	1.115	0.092	0.38	1.732	0.000	1.72
	Low	1.145	0.011	0.51	1.276	0.007	1.18	0.955	0.438	-0.18	1.303	0.005	0.70
Time to PhD degree		1.015	0.079	0.27	1.024	0.156	0.56	0.982	0.241	-0.31	1.028	0.055	0.35
Involvement in research groups (reference: no)		0.797	0.000	-0.08	0.945	0.615	-0.03	0.830	0.042	-0.07	0.705	0.003	-0.07
Collaboration with the supervisor (reference: no)		0.883	0.001	-0.13	0.860	0.005	-0.21	0.953	0.443	-0.06	0.847	0.011	-0.11
University service (reference: no)		0.988	0.698	-0.03	0.939	0.136	-0.22	1.044	0.315	0.14	0.964	0.731	-0.04
Inbred status (reference: no)		0.859	0.002	-0.23	0.953	0.807	-0.10	0.902	0.174	-0.20	0.691	0.000	-0.32
Place of PhD (reference: Spain)		1.055	0.607	0.29	1.090	0.846	0.70	0.934	0.612	-0.39	1.084	0.417	0.28
Postdoctoral international mobility (reference: no)	Yes, short stay (<6 months)	1.089	0.051	0.37	1.062	0.815	0.36	1.110	0.096	0.49	1.054	0.393	0.14
	Yes, long stay	1.209	0.014	0.79	1.082	0.136	0.40	1.289	0.024	0.99	1.319	0.876	0.94
Mobility across research groups (reference: no)		1.110	0.001	0.43	1.074	0.092	0.38	1.076	0.093	0.31	1.212	0.004	0.58
Mobility outside academia (reference: no)		1.333	0.000	1.75	1.048	0.657	0.35	1.640	0.000	3.71	1.489	0.011	1.60
Sex (reference: women)		0.960	0.157	-0.07	1.042	0.306	0.14	0.899	0.012	-0.21	1.008	0.687	0.01
Age at PhD		0.959	0.000	-0.82	0.967	0.023	-0.81	0.976	0.036	-0.48	0.948	0.000	-0.71
Research field (reference: Biology and Biomedical Sciences)	Exact and Natural Sciences	0.742	0.000	-0.94	-	-	-	-	-	-	-	-	-
	Engineering and Technological Sciences	0.503	0.000	-2.00	-	-	-	-	-	-	-	-	-
Research field × Postdoctoral Publications (PPUB) by-year (ln) (reference: Biology and Biomedical Sciences × PPUB)	Exact and Natural Sciences × PPUB	1.090	0.028	0.34	-	-	-	-	-	-	-	-	-
	Engineering and Technological Sciences × PPUB	1.180	0.000	0.54	-	-	-	-	-	-	-	-	-
Research orientation of the university granting the tenure (reference: High)	Medium	0.922	0.059	-0.29	0.943	0.364	-0.29	0.982	0.800	-0.07	0.684	0.000	-0.79
	Low	0.887	0.015	-0.40	0.898	0.130	-0.49	1.016	0.766	0.06	0.701	0.000	-0.73
Labor market (reference: Low)	Medium	0.932	0.055	-0.26	0.900	0.049	-0.57	0.915	0.092	-0.37	1.048	0.500	0.11
	High	0.828	0.000	-0.64	0.813	0.002	-1.19	0.881	0.021	-0.46	0.832	0.019	-0.34
Constant		34.33	0.000		20.473	0.000		16.316	0.000		29.236	0.000	
Time at risk		7256			2835			2786			1635		
Number of subjects		1257			371			463			423		
Number of failures		1257			371			463			423		
Log Likelihood		-912.1			-195.1			-291.7			-360.1		
LR Chi-square		718.3			96.41			164.21			153.97		
Prob>Chi-square		0.000			0.000			0.000			0.000		
Akaike		1875			432.7			625.9			762.8		
Natural logarithm of gamma		-1.3			-1.48			-1.39			-1.15		
Expected general duration				**5.45**			**7.74**			**5.97**			**3.36**

Examining the direction of the effects on promotion of the set of variables that we have considered as measures of performance, productivity, or academic potential, we find, on the one hand, that the more an academic has published before earning a PhD (early publication), the faster he/she advances in his/her career, with a reduction in duration of more than 10 months (-0.86 years). On the other hand, in this model, we observe that postdoctoral productivity is not significant. The effect of the research orientation of the university granting the degree goes in the expected direction: those having a degree from universities with a stronger research orientation advance faster and obtain tenure earlier. Another indicator of past performance, namely, time to PhD completion, is barely significant, increasing time to tenure by a little over 3 months (0.27 years). In terms of the effects of variables indicating social embeddedness, they are all associated with a reduction of time to tenure. Collaborating with a PhD supervisor during the period preceding tenure and carrying out research activity mainly in the context of research groups, as opposed to not belonging to groups, both accelerate promotion and reduce the duration of the non-tenure situation. Involvement in institutional service is not statistically significant, but inbred status reduces time to tenure. In general, the size of the effects of the social embeddedness variables are small; for example, collaboration with the PhD supervisor after obtaining the PhD accelerates career advancement by not quite a month and half (-0.13 years) while involvement in research groups does so by less than a month (-0.08 years) with respect to those who are not involved. The exception is inbred status, which advances a career by almost 3 months (-0.23 years).

Regarding the effects of mobility variables on time to tenure, the results show that all forms of mobility affect time to tenure negatively. Having obtained a PhD abroad is not statistically significant, but having experienced international mobility as a part of the postdoc and having taken a job in a non-academic sector increase the duration. A further form of mobility related to changing research groups also extends duration. Moreover, the size of the effects is relevant. The effect of international mobility in the postdoctoral period for those who experienced such mobility, in comparison with the group that did not, is clearly detrimental. For the group having short stays (less than 6 months), the effect increases time to tenure by more than 4 months (-0.37 years) while for the group with longer stays, the delay is more than 9 months more (-0.79 years). Mobility between different research groups produces a delay of almost 5 months (0.43 years). However, the worst effect of mobility on an academic career arises from intersectorial mobility to a non-academic job, which increases time to tenure by 1 year and 9 months (1.75 years).

Regarding the control variables, despite the differences in duration by sex, when the effects of other variables are introduced into the model, we cannot find significant effects. Considering previous findings in the literature about gender gaps, we built up independent models for males and females and found that both models have the same direction of the effects of the covariates. The effect of age at PhD is significant: as age at PhD increases, the duration of the period until tenure diminishes, indicating that some seniority or age effects reduce time to tenure once the PhD degree is obtained. When age at PhD increases by more than three-and-a-half years (one SD), the length of time elapsed to tenure is reduced by almost 10 months (-0.82 years).

There is also significant diversity in time to tenure by scientific fields. For faculty from engineering and technological fields, it takes 2 years less to obtain tenure than for academics from Biology and Biomedical Sciences; for faculty from Exact and Natural Sciences, the difference is more than 11 months (-0.94 years) less. We also ran independent models (log-logistics) separately for the three research fields and found that for all of the variables that stay statistically significant at the level p < 0.05, there is no change in the direction of the effects of the variables. However, almost half of the variables lose significance (Table 6).

Regarding the interaction between scientific fields and postdoctoral publications, the results reveal that it is in the Engineering and Technological fields where the model of career is more decoupled from postdoctoral publications. The effect of the research orientation of the university granting tenure is significant only for the less research-oriented universities, which speed up advancement in rank. Achieving tenure at less research-oriented universities reduces duration by 5 months (-0.40 years) compared to getting tenure at universities with high research orientation. Finally, the effect of the demand level or growth of the universities granting tenure, as a measure of the dynamism of the labor markets, is an important variable to account for the duration: obtaining tenure at universities with high rates of growth reduces duration by more than 8 months (-0.64 years) in comparison with universities with a low level of position growth.

Considering the relevance of the effect on international mobility, in Table 7 we present the estimates of the duration of the log-logistic model for each of the 3 groups regarding the international mobility experience variable. To control the potential effect of time out resulting from the international mobility experience of the candidates we have run the log logistics model for the three categories we have regarding international mobility. While the direction of the effects of the covariates does not change among the three groups, we observe a significant increase in the expected general duration (EGD) for the mobile groups. While for those that did not have any international experience the EGD was 4.68 (4 year and 8 months) for the group of individuals with less than 6 months of postdoctoral experience the increase in EGD was 5.41 (5 years and 5 months), meaning 7 months more than the other group. For those with 6 months or more of international postdoctoral experience the EGD was almost 7 years (2 years and 4 months more). After checking the average durations for the groups we can say that international mobility does decelerate time to tenure in the Spanish system. In terms of early tenure and fast career advancement international mobility seems not to pay; however in the long term it could provide benefits to those with the experience or increase the productivity of the whole system, something that should be tested in further empirical research. It is also interesting to observe what we could call the accumulated effect of long international mobility experience and mobility outside academia. For the group who moved outside academia the number increases by 4 additional years.

**Table 7:** Log-logistic survival model and expected length of time to tenure by type of Postdoctoral international mobility.

		**General**			**Postdoctoral international mobility: No**			**Postdoctoral international mobility: Yes, short stay (<6 months)**			**Postdoctoral international mobility: Yes, long stay.**		
**Variables**		**Time ratio**	**P>|z|**	**Diff (years).**	**Time ratio**	**P>|z|**	**Diff (years).**	**Time ratio**	**P>|z|**	**Diff (years).**	**Time ratio**	**P>|z|**	**Diff (years).**
Early publications (ln)		0.783	0.000	-0.86	0.808	0.000	-0.66	0.838	0.002	-0.70	0.736	0.000	-1.17
Postdoctoral publications by-year (ln)		0.985	0.604	-0.07	0.967	0.501	-0.14	0.992	0.901	-0.04	1.015	0.697	0.09
Research orientation of the university granting the PhD (reference: High)	Medium	1.216	0.000	0.71	1.319	0.000	0.94	1.323	0.002	1.12	1.009	0.882	0.03
	Low	1.145	0.011	0.51	1.224	0.014	0.70	1.198	0.069	0.60	0.928	0.347	-0.34
Time to PhD degree		1.015	0.079	0.27	1.027	0.034	0.49	1.020	0.248	0.31	0.958	0.016	-1.41
Involvement in research groups (reference: no)		0.797	0.000	-0.08	0.726	0.001	-0.10	0.874	0.333	-0.03	0.909	0.261	-0.04
Collaboration with the supervisor (reference: no)		0.883	0.001	-0.13	0.810	0.000	-0.18	1.022	0.778	0.02	0.883	0.007	-0.19
University service (reference: no)		0.988	0.698	-0.03	1.003	0.953	0.00	0.950	0.378	-0.10	1.004	0.916	0.02
Inbred status (reference: no)		0.859	0.002	-0.23	0.853	0.007	-0.20	0.718	0.000	-0.50	1.012	0.820	0.03
Place of PhD (reference: Spain)		1.055	0.607	0.29	1.065	0.764	0.30	0.951	0.811	-0.26	1.049	0.745	0.34
Postdoctoral international mobility (reference: No)	Yes, short stay (<6 months)	1.089	0.051	0.37	-	-	-	-	-	-	-	-	-
	Yes, long stay	1.209	0.014	0.79	-	-	-	-	-	-	-	-	-
Mobility across research groups (reference: no)		1.110	0.001	0.43	1.132	0.027	0.49	1.044	0.523	0.18	1.114	0.007	0.46
Mobility outside academia (reference: no)		1.333	0.000	1.75	1.331	0.011	1.48	1.266	0.164	1.39	1.602	0.002	4.08
Sex (reference: women)		0.960	0.157	-0.07	1.019	0.702	0.03	0.866	0.024	-0.22	0.967	0.390	-0.08
Age at PhD		0.959	0.000	-0.82	0.949	0.000	-0.95	0.957	0.002	-0.77	0.998	0.847	-0.12
Research field (reference: Biology and Biomedical Sciences)	Exact and Natural Sciences	0.742	0.000	-0.94	0.657	0.000	-1.22	0.705	0.001	-1.06	0.864	0.025	-0.48
	Engineering and Technological Sciences	0.503	0.000	-2.00	0.486	0.000	-1.48	0.459	0.000	-2.24	0.635	0.000	-2.29
Research field × Postdoctoral Publications (PPUB) by-year (ln) (reference: Biology and Biomedical Sciences x PPUB)	Exact and Natural Sciences × PPUB	1.090	0.028	0.34	1.111	0.108	0.31	1.091	0.252	0.36	1.035	0.482	0.18
	Engineering and Technological Sciences × PPUB	1.180	0.000	0.54	1.126	0.046	0.36	1.225	0.010	0.79	1.094	0.221	0.27
Research orientation of the university granting the tenure (reference: High)	Medium	0.922	0.059	-0.29	0.836	0.016	-0.55	0.835	0.028	-0.62	1.127	0.037	0.53
	Low	0.887	0.015	-0.40	0.822	0.011	-0.56	0.778	0.006	-0.78	1.159	0.034	0.69
Labor market (reference: Low)	Medium	0.932	0.055	-0.26	0.947	0.352	-0.17	0.923	0.290	-0.31	0.918	0.090	-0.41
	High	0.828	0.000	-0.64	0.824	0.002	-0.54	0.774	0.001	-0.80	0.900	0.053	-0.52
Constant		34.33	0.000		52.138	0.000		41.123	0.000		12.696	0.000	
Time at risk		7256			2973			1727			2556		
Number of subjects		1257			600			296			361		
Number of failures		1257			600			296			361		
Log Likelihood		-912.1			-494.0			-245.4			-145.8		
LR Chi-square		718.3			309.7			182.8			148.1		
Prob>Chi-square		0.000			0.000			0.000			0.000		
Akaike		1875			1035			457			338		
Natural logarithm of gamma		-1.3			-1.2			-1.3			-1.6		
Expected general duration				**5.45**			**4.68**			**5.41**			**6.97**

In Table 8 we reproduce the results considering the postdoctoral publications as a time-varying covariate (TVC), to deal with the potential problems of endogeneity and interpretation. There are not big differences with the results of most of the other variables included in Table 6, but when checking the TVC we see that, being significant, the effect of postdoctoral publications interacting with time is one of reduction of duration (-0.168). We also observe that there is a significant increase in duration associated with the interactions between annual postdoctoral publications and the fields of Experimental Sciences and Engineering (with regards to the reference category) (0.180). It is important to note that the interaction of postdoctoral publications with time becomes significant and yields results in the sense that the more a scholar has published after the PhD the shorter the duration of time to tenure; this confirms that postdoctoral publications, when treated as TVC, positively affect the duration of time to tenure.

**Table 8:** Log-logistic survival model with time-varying covariate (TVC).

	**Variables**		**Coeff.**	**P>|z|**
**Main**				
	Early publications (ln)		-0.250	0.000
	Postdoctoral publications by-year (ln)		0.029	0.290
	Research orientation of the university granting the PhD (reference: High)	Medium	0.184	0.000
		Low	0.133	0.009
	Time to PhD degree		0.013	0.118
	Involvement in research groups (reference: no)		-0.216	0.000
	Collaboration with the supervisor (reference: no)		-0.145	0.000
	University service (reference: no)		-0.013	0.627
	Inbred status (reference: no)		-0.133	0.000
	Place of PhD (reference: Spain)		0.058	0.587
	Postdoctoral international mobility (reference: no)	Yes, short stay (<6 months)	0.084	0.016
		Yes, long stay	0.169	0.000
	Mobility across research groups (reference: no)		0.101	0.001
	Mobility outside academia (reference: no)		0.286	0.000
	Sex (reference: women)		-0.041	0.163
	Age at PhD		-0.040	0.000
	Research field (reference: Biology and Biomedical Sciences)	Exact and Natural Sciences	-0.271	0.000
		Engineering and Technological Sciences	-0.662	0.000
	Research field × Postdoctoral Publications (PPUB) by-year (ln) (reference: Biology and Biomedical Sciences x PPUB)	Exact and Natural Sciences × PPUB	0.057	0.100
		Engineering and Technological Sciences × PPUB	0.122	0.002
	Research orientation of the university granting the tenure (reference: High)	Medium	-0.064	0.123
		Low	-0.105	0.024
	Labor market (reference: Low)	Medium	-0.081	0.022
		High	-0.185	0.000
	Constant		3.437	0.000
**Time-Varying Covariates (Natural logarithm of gamma)**				
	Postdoctoral publications (PPUB) -by-year (ln) × time		-0.168	0.000
	Research field × Postdoctoral Publications (PPUB) by-year (ln) × time (reference: Biology and Biomedical Sciences x PPUB × time)	Exact and Natural Sciences × PPUB × time	0.041	0.369
		Engineering and Technological Sciences × PPUB × time	0.180	0.000
	Constant × time		-1.198	0.000
	Time at risk		7256	
	Number of subjects		1257	
	Number of failures		1257	
	Log Likelihood		-898.6	
	LR Chi-square		697.61	
	Prob>Chi-square		0.000	
	Akaike		**1848**	

Finally, as an additional control of the whole set of results, we have replicated the analysis with a semi-parametric Cox model, with time-varying covariate. Results are presented in Table S5 in File S1, including the hazard rate. This Cox model also provides confirmation of the robustness of the analysis and interpretation and confirms the need to take into account the TVC and their interactions with fields, also confirming the results of our log logistics model. In Table S6 in File S1and Table S7 in File S1we present the Cox model by research fields and by international mobility experience.

## Discussion

This paper has explored the process of transition to tenure in academia to address the issues related to time to permanent employment at Spanish universities. After trying four parametric models and one semi-parametric model, we found that the better fit to our data is provided by a log-logistic model in which the rate of transition increases monotonically at the beginning and then starts to drop, producing an inverted U shape. Interestingly, the promotion process in Spain can be modeled as a diffusion process. The most relevant variables associated with time to tenure refer to mobility; the negative effects of all kinds of mobility on time to tenure not only provide negative incentives for researchers to change jobs, organizations, or countries but are also a clear expression of the absence of open academic job markets and the existence of mechanisms for accessing the profession that could be shaped by particularistic dynamics. In addition, a promotion system characterized by accelerated tenure could be grounded on the construction of research teams and on a strategy of commitment developed by the departments (which control the hiring process but not the provision of new positions) as a way to cope with the rigidities and risks (uncertainties) of the hiring process.

Our results in terms of the role of past academic performance provide some support for the claims of feedback or cumulative effects on careers as far as publications prior to the PhD are concerned [34]. Our postdoctoral productivity measure, when considered as a time varying covariate, is also associated with a reduction of the duration of the transition.

Obtaining the PhD at a highly research oriented university is positively related to timing and to fast advancement of careers. This represents a feature of a system in the process of growing differentiation in terms of stratification and reputation.

Finally, a traditional indicator a candidate’s research potential, such as time to PhD, produces minor differences; when we consider that age at PhD reduces transition time, we could presume that there are informal “queues” at universities based on time since entry and not on timing of the PhD graduation, generating an informal seniority reward model among tenure candidates. Usually, academics finishing their PhD at an older age and having faster promotion have been teaching, but their entry into the department would have taken place quite early after their bachelor’s degree. Implicit contracts exist between the PhD candidate and the PhD supervisor, mutual expectations about collaboration and recruitment that occur in the research training period [83]. Thus, the entry into the labor market often takes place very early in the research career, and this period is more strongly associated with learning rather than with demonstrating performance. In our population, the average age of entry in the institution that granted the permanent position was 28 years, while the average age at PhD was almost 31, and the average age at tenure was 36. All of these factors could indicate the existence of some seniority rules that might be in place in Spanish academia, accelerating career advancements as you become older or senior, as it is also the case of universities in Canada [18].

Some results from our study suggest the existence of internal academic labor markets as a way of protecting investments and competing with the outside world (when higher salaries and more differentiation exist). First, seniority helps determine the timing of promotion, indicating the existence of informal “queues” within the departments and that, as age at earning the PhD increases, the length of time to tenure diminishes. Second, inbreeding is a significant predictor of reduction of time to tenure, and when we model independently academics in the engineering and technological areas, inbred status accelerates the advancement of careers even more. Thus, different areas of research have different career models, as can be deduced from the very heterogeneous length of time to tenure among researchers in our three different fields. Engineers have faster career tracks, probably because of the greater difficulties that departments face in recruitment thanks to external employment opportunities. Academics in the areas of Natural and Exact Sciences also have much faster career advancement than those in the Biological and Biomedical Sciences.

The major predictors of fast access to a permanent job are related to absence of mobility: international, sectorial, or even cross-research group mobility implies longer time to promotion. Even discounting the time individuals have been abroad, internationally mobile scholars take more time to tenure than their nonmobile counterparts. We have found an academic system averse to mobility, probably related to the lack of control by the departments regarding the creation of new positions; the risk of losing the position if people leave the university leads them to prefer loyalty and penalize mobility.

It is interesting to note that the effect of the mobility variables is far more important, in terms of size, than those measuring academic social embeddedness and productivity. If the first set of factors indicates where candidates are or have been before tenure and the second set indicates what they have done (mentor collaboration or group involvement, publications), we have to infer that “staying” is more relevant. We conclude that some particularistic factors could be in operation in the process of obtaining a permanent job in academia in the case under study. It seems that career advancement in the Spanish university system, in aggregate terms, while grounded in a merit-based system, also has elements associated with the integration and permanence of the candidates in their local academic environments to promote productive teamwork.

Despite the present low level of differentiation in the Spanish university system, earning a permanent position was faster in universities scoring low in the ranking of research orientation. There might be a self-selection mechanism at work; however, the results are consistent with some previous findings [75] that graduates from less research-oriented universities applied for tenure at less research-oriented universities, which were less demanding with the recruitment and provide faster promotion than research-intensive universities.

Our findings indicate that the level of demand in the universities providing new positions is an important factor for analysis of time to tenure. The context of the academic labor market is relevant [67]: Candidates in high-growth universities experience lower duration. Spanish universities have been growing in faculty at very diverse rate. Their budgetary dependence on 17 different regional governments has created this conditional frame. Reforms regarding the management of tournaments for access to a permanent academic position have been implemented since 2001, first as a change into a national accreditation exams system and later on as a transition to an accreditation system based on CV evaluation, creating a pool of accredited academics, from among which universities could select candidates for new tenure positions.

Life employment (civil servant status) in this context is also part of a feedback mechanism; once the academic is granted tenure, he/she becomes institutionally “trapped” with little chance of changing universities without going again through a formal tournament (exam). Individuals must therefore make early decisions about where and in which university/institutions they want to spend their professional careers; in this institutional context, rational actors could decide where they would like to stay and wait in temporary positions until they get tenure, even if this is slower than taking the risk of mobility.

Our analysis of promotion found no significant differences in the timing of promotion for women. This result is in line with some recent work reporting that career trajectories of men and women are converging at junior levels (access to associate professor) and that men have only a very slight or non significant advantage in terms of first promotion [18,86]. One interesting element when we run independent models for males and females is the fact that university service was significant in the case of males and contributed to the reduction of time in getting a permanent job; this result is consistent with findings regarding a research institution in France [30].

Our findings suggest the existence of diverse models of relationships among mobility, merit, and academic promotion and render questionable the argument that mobility is rewarded. In some systems, it seems that having a stable, low-mobile postdoctoral trajectory is much more important than the place of PhD training in terms of having a faster access to a permanent position. Temporary mobility in careers could come after tenure. Institutions and their strategies have a key role in shaping what is valued in each moment of a career track and for some institutions.

On the methodological side, event history analysis has proven to be quite robust for modeling the duration and the timing of academic promotion and its explanatory factors, an analysis that is key in understanding the functioning of systems based on low mobility and relative early tenure. On the policy side, it is difficult to reconcile the emphasis placed on the desirability of mobility as a way of assuring knowledge circulation and research collaboration, with the evidence that it is negatively associated with the duration of transition to tenure.

We have to acknowledge that the system of recruitment and access to tenure is based mainly on promises and expectations. However, once the academic is inside, he/she has life employment. The question of how individuals who are aware of the incentive system that really exists can be mobilized to bring excellence to the organizations that hire them remains open.

This study employed a research design that could overcome some of the weaknesses of previous studies, indicators of potential generalization of results to systems with similar institutional arrangements like Mexico [87], Portugal [88], France [30], Italy [68], and Canada [18,29]. However, some caveats should be acknowledged. First, we have concentrated here on what we consider the first major career transition in the academy: promotion to associate professorship. The length of time to promotion to full professor positions might be affected by additional factors, or by the factors here analyzed in a different way [89]. Second, our data refers to academics who got tenure prior to 2002. In the last decade, university reform laws have changed recruitment and appointment procedures. Additionally, public research organizations have faced some pressures to show strong research performance. The effect of these changes on promotion processes is an issue that deserves further research. Third, although we have characterized universities with different situations, we have analyzed universities in an aggregated way; further research and analysis of individual universities and fields is needed to measure the effects of different practices and policies.

## Supporting Information

File S1 **Tables S1-S7.** Table S1, Institutional features of the public university system in Spain: a snapshot. Table S2, Description of the variables. Table S3, Correlations. Table S4, Time to obtaining a permanent academic job, parametric and non-parametric models. Table S5, Cox semiparametric model with time-varying covariates. Table S6, Cox semiparametric model with time-varying covariate by Research Field. Table S7, Cox semiparametric model with time-varying covariate by type of International Mobility.
(DOCX)

File S2 **Questionnaire.**
(PDF)

File S3 **Annex: Model specification.**
(DOC)
