# Supplementary material for: Time to Tenure in Spanish Universities: An Event History Analysis
Source: PLoS One. 2013 Oct 8;8(10):e77028. doi: 10.1371/journal.pone.0077028 (PMC3792917; doi:10.1371/journal.pone.0077028)
Supplement: File S1 — Tables S1-S7. Table S1, Institutional features of the public university system in Spain: a snapshot. Table S2, Description of the variables. Table S3, Correlations. Table S4, Time to obtaining a permanent academic job, parametric and non-parametric models. Table S5, Cox semiparametric model with time-varying covariates. Table S6, Cox semiparametric model with time-varying covariate by Research Field. Table S7, Cox semiparametric model with time-varying covariate by type of International Mobility. (DOCX) [file pone.0077028.s001.docx]

**Table S1. Institutional features of the public university system in Spain: a snapshot**

| **Basic Features** | **Description** |
| --- | --- |
| **Publicness** | The Spanish university system is similar to other European systems like those of France, Italy, and Germany, where public universities account for the greatest share of the system. For a more recent and detailed comparative analysis, see [10]. |
| **Governance and autonomy** | It is a state-led model managed by professional academic corporations, constitutionally autonomous from government but subjected to public sector rules as regards budgeting, human resources management, contracting, etc. |
| **Funding** | Universities are financially dependent on regional governments. On average, direct transfers represent up to ¾ of the total budget; the rest comes from student fees and research activities. |
|  | Direct funding is not provided on the basis of performance [11] but mainly on the number of students and type of degree. |
|  | While direct funding is not earmarked as mandatory, the universities have to guarantee the civil servant salaries (faculty and staff) first. |
|  | Universities have a very limited need to compete for the best professors to keep going or to advance financially because the contribution of research overhead to their overall funding is small. |
| **Status of academic staff** | The university academic employment structure is dual: Temporary professors or researchers working on fixed-term contracts, under lectureships, PhD fellowships, or temporary contracts for research projects. One of these positions is usually the entry job in the department, which is occupied early in the career. Mutual expectations about future permanence often develop. Permanent (tenured) professors (with civil servant status and life employment guaranteed by the State) after winning a public “tournament” [12]. |
|  | There are no positions under the “tenure track” model, as a probationary system for a fixed-term period, after which the subjects are “up or out.” |
| **Capacity of departments to manage human resources and positions** | Departments have some capacity over the creation of temporary positions but very little over the creation of new permanent ones, a function highly centralized in university authorities, who are democratically elected. |
|  | Departments or institutes do not manage their positions in an autonomous way to fulfill objectives. |
|  | If a permanent faculty member leaves the institution, the position is often completely lost for the department and new rounds of negotiations with authorities start from scratch to try to get a new position in competition with other units at the university. |
|  | This context of authority distribution creates some pressure for the department to support and reward “loyal candidates” who will not leave or go away; in doing so, departments minimize the risk of losing positions that are costly to get. |
|  | There are no salary negotiations in academic recruitment. In Spain, as in many other European countries, permanent university faculty members are civil servants, and their salaries are established on the basis of bureaucratic rules. This feature limits the negotiating capacity of departments and their ability to motivate their members once recruited. |
|  | Evaluation of performance in research and teaching is poorly developed and set up in a national exercise; it has only positive consequences for those well evaluated, with a very small increase of salary [13]. |
| **Tournament call and selection procedure for tenure** | The creation of a new permanent position has to be approved by the university central administration and is allocated to the department after a complex political negotiation, usually based on the average number of students per faculty in the different schools. |
|  | In 2001, the year of reference for access to tenure of our study, the way of filling out the new permanent position was a public call for a tournament. All PhDs could apply and compete in a quasi-public exam system. The composition of the examining committee, at the time of the study, was determined by a national law (5 members: the chair and one member proposed by the department, and 3 other members selected by a lottery among the tenured professors in the field of the research subject of the position, working in any Spanish public university). |
|  | Applicants might be already working in the department or come from outside, but the system implies high transaction costs for those not in the department because academic cultures tend to value loyalty highly and tend to reward it accordingly. |
| **Decision-making and incentive structure** | Life employment (civil servant status) in this context is also part of a feedback mechanism; once the academic is granted tenure, he/she becomes institutionally “trapped” with no chances of changing universities without again going through a formal tournament (exam). |
|  | Individuals make early decisions about where and in which university/institutions they want to spend their professional careers adapting to the incentive structure. |
|  | In this institutional context, rational actors could decide where they would like to stay and wait in temporary positions until they get tenure, even if this choice is slower than taking the risk of mobility. |

Source: Authors’ elaboration

**Table S2. Description of the variables**

| **Factors** | **Variable** | **Description** | | **Sources and operationalization:** | |
| --- | --- | --- | --- | --- | --- |
|  | **Time to tenure** | **Number of years elapsed between doctoral degree and tenure** | | **Questionnaire. Comparison between the answer to 2 questions: year of obtaining the PhD and year of tenure** | |
| **Academic performance** |  | |  |  | |
|  | Postdoctoral publications *(from PhD until tenure)* | A quantitative variable representing the individual annual scientific productivity between the year of PhD and the year of tenure | | ISI Web of Science. All types of documents. Whole counting system. Represents the number of times the name of an individual appears as author in papers, regardless of the number of co-authors, every year since doctoral degree until the year of getting tenure (1). | |
|  | Early publications *(before phD)* | A quantitative variable representing the individual annual scientific productivity during or before the year they were awarded the PhD | | ISI Web of Science. All types of documents. Whole counting system. Represents the number of times the name of an individual appears as author in papers, regardless of the number of co-authors, divided by the number of years elapsed since doctoral degree (1). | |
|  | Research orientation of the university granting the PhD | Categorical variable that classifies individual universities where the academic earned the PhD, according to their success rates in obtaining competitive funding (2) | | Administrative data. The categories are high (reference), medium, and low. The tiers include the 39 cases from 8, 11, and 20 Spanish PhD-granting universities (3). | |
|  | Time to PhD degree | Quantitative variable calculated to represent the number of years elapsed between getting the bachelor’s degree and getting the PhD; used as a proxy of the rate of educational progress. | | Questionnaire. Comparison between the answer to 2 questions (year of obtaining the PhD and year of earning the bachelor’s degree) | |
| **Social embeddedness** |  |  | |  | |
|  | Involvement in research groups | Dummy variable measuring whether or not the individual has carried out activity in the postdoc period in the context of research groups or teams | | Questionnaire. Codification based on a question asking if the PhD has carried out his/her research activity in the context of teams | |
|  | Collaboration with PhD supervisor | Dummy variable measuring whether or not the respondent has continued collaborating in research with their PhD supervisor after earning the PhD | | Questionnaire. Codification based on a question asking if the PhD has continued collaborating in research with their PhD supervisor after getting their doctoral degree | |
|  | University service | Dummy variable measuring whether or not the respondent has been involved in representative, administrative, managerial, or bureaucratic positions in the university after the PhD | | Questionnaire. Codification based on a question (multiple answers possible) asking whether or not the respondent has been involved in at least one different type of university service: representative, administrative, managerial, or bureaucratic positions (school board representation, vice-deans, deputy or secretary of the department, etc.) in the university after the PhD | |
|  | Inbreeding status | Dummy variable measuring whether or not the individual obtained tenure at a different university from the one that awarded him/her the PhD degree (4) | | Questionnaire. Comparison between the answer to 2 (university granting degree and university granting tenure) | |
| **Mobility** |  |  | |  | |
|  | PhD in a foreign university | Dummy variable that measures whether or not the individual has been granted the PhD by a foreign university | | Questionnaire. Codification based on the question of university and country of granting the PhD | |
|  | Postdoctoral international mobility | Categorical variable that measures if the respondent reports academic postdoctoral stays abroad (of more than 1 month) | | Questionnaire. Codification based on 2 questions asking if the respondent reports academic postdoctoral stays abroad and for how long; three categories for comparison: no stays (reference), short-term visit (less than six months) and long term stay. | |
|  | Mobility across research groups | Dummy variable measuring whether or not the individual has worked with more than 1 research group in the postdoc period | | Questionnaire. Codification based on a question asking if the PhD has been working during his/her postdoctoral research activity with more than 1 research team | |
|  | Mobility outside academia | Dummy variable measuring whether or not the respondent obtained their first post-PhD employment in a non-academic or non-research organization | | Questionnaire. Codification based on a question asking for the sector of the first employment after the PhD | |
| **Control variables** |  |  | |  | |
|  | Age at PhD | Quantitative variable representing the age of individuals at the time of getting PhD | | Questionnaire. Direct question | |
|  | Sex | Male or female | | Questionnaire. Direct question | |
|  | Research field | Categorical variable of the researcher’s main domain of activity | | Questionnaire. Codification of the area of the PhD diploma according the OECD Fields of Science Classification at field level. The 3 possible values are Biology and Biomedical Sciences (used as a reference in the models); Exact and Natural Sciences; and Engineering and Technological Sciences. | |
|  | Research orientation of the university granting tenure | Categorical variable that classifies individual universities where the academic earned tenure, according to their success rates in getting competitive funding (2) | | Administrative data. The categories are high, medium, and low. The tiers include cases from a total of 37 universities granting tenure: 10, 12, and 15 in each tier. | |
|  | University demand level | Categorical variable that classifies individual universities in which the academic earned tenure according to the average aggregate rate level of growth in new tenured positions in the years under consideration (1997–2001) | | Administrative data. The 3 categories are low growth (below the average growth of the university sector), medium growth (up to 50% more than the national average), and high growth (more than 50%). Categories included 37 universities with 13, 12, and 12 in each tier (5). | |

***Explanatory Notes:***

(1) This variable was transformed into its natural logarithm because of the skewness of the distribution.

(2) Because in Spain there is no recognized ranking of reputation, we used this proxy of university “research orientation” to stratify the universities.

The tiers are constructed based on the ranking of research competitiveness [14], which represents the position of each university with regard to the ratio of success in applications for competitive funding of R&D projects.

(3) All PhDs obtained in non-Spanish universities have been included in the high research orientation group, as a single group category, based on the standard assumption of higher reputational effects [28].

(4) It includes the so-called “silver corded” scholars [16].

(5) Average rate of growth, in 1997 to 2001, in the number of associate professors in Spanish public universities was 56%.

**Table S3. Correlations**

| **Number** | **Variable** | **Variable** | **1** | **2** | **3** | **4** | **5** | **6** | **7** | **8** | **9** | **10** | **11** | **12** | **13** | **14** | **15** | **16** | **17** | **18** | **19** | **20** | **21** | **22** | **23** |
| --- | --- | --- | --- | --- | --- | --- | --- | --- | --- | --- | --- | --- | --- | --- | --- | --- | --- | --- | --- | --- | --- | --- | --- | --- | --- |
| 1 | **Time to Tenure** |  | 1 |  |  |  |  |  |  |  |  |  |  |  |  |  |  |  |  |  |  |  |  |  |  |
| 2 | **Early publications (ln)** |  | -0.20 | 1 |  |  |  |  |  |  |  |  |  |  |  |  |  |  |  |  |  |  |  |  |  |
| 3 | **Postdoctoral publications -by-year (ln)** |  | 0.10 | 0.62 | 1 |  |  |  |  |  |  |  |  |  |  |  |  |  |  |  |  |  |  |  |  |
|  | **Research orientation of the university granting the PhD (reference: high)** |  |  |  |  |  |  |  |  |  |  |  |  |  |  |  |  |  |  |  |  |  |  |  |  |
| 4 |  | Medium | 0.10 | -0.03 | 0.01 | 1 |  |  |  |  |  |  |  |  |  |  |  |  |  |  |  |  |  |  |  |
| 5 |  | Low | -0.05 | 0.06 | 0.01 | -0.61 | 1 |  |  |  |  |  |  |  |  |  |  |  |  |  |  |  |  |  |  |
| 6 | **Time to PhD degree** |  | -0.04 | -0.15 | -0.07 | 0.04 | -0.10 | 1 |  |  |  |  |  |  |  |  |  |  |  |  |  |  |  |  |  |
| 7 | **Involvement in research groups (reference: no)** |  | -0.06 | 0.09 | 0.04 | 0.01 | 0.10 | -0.17 | 1 |  |  |  |  |  |  |  |  |  |  |  |  |  |  |  |  |
| 8 | **Collaboration with the supervisor (reference: no)** |  | -0.08 | 0.11 | 0.02 | -0.01 | 0.13 | -0.07 | 0.18 | 1 |  |  |  |  |  |  |  |  |  |  |  |  |  |  |  |
| 9 | **University service (reference: no)** |  | -0.04 | -0.05 | -0.07 | -0.11 | 0.05 | 0.09 | 0.09 | 0.07 | 1 |  |  |  |  |  |  |  |  |  |  |  |  |  |  |
| 10 | **Inbred status (reference: no)** |  | -0.05 | 0.08 | 0.05 | 0.04 | 0.05 | -0.01 | 0.17 | 0.18 | 0.03 | 1 |  |  |  |  |  |  |  |  |  |  |  |  |  |
| 11 | **Place of PhD (reference: Spain)** |  | 0.00 | -0.02 | 0.02 | -0.12 | -0.10 | -0.06 | -0.11 | -0.13 | -0.08 | -0.21 | 1 |  |  |  |  |  |  |  |  |  |  |  |  |
|  | **Postdoctoral international mobility (reference: no)** |  |  |  |  |  |  |  |  |  |  |  |  |  |  |  |  |  |  |  |  |  |  |  |  |
| 12 |  | Yes, short stay (<6 months) | 0.01 | 0.04 | 0.05 | -0.04 | 0.05 | -0.02 | 0.05 | 0.05 | 0.08 | -0.02 | -0.01 | 1 |  |  |  |  |  |  |  |  |  |  |  |
| 13 |  | Yes, long stay | 0.07 | 0.05 | 0.06 | 0.09 | -0.01 | -0.28 | 0.02 | -0.04 | -0.22 | -0.01 | 0.03 | -0.41 | 1 |  |  |  |  |  |  |  |  |  |  |
| 14 | **Mobility across research groups (reference: no)** |  | 0.05 | 0.02 | 0.01 | 0.02 | 0.00 | -0.03 | 0.19 | 0.00 | 0.04 | -0.07 | 0.03 | -0.04 | 0.18 | 1 |  |  |  |  |  |  |  |  |  |
| 15 | **Mobility outside academia (reference: no)** |  | 0.13 | -0.09 | -0.06 | 0.07 | -0.05 | 0.00 | -0.14 | -0.01 | -0.06 | -0.03 | -0.01 | 0.01 | -0.04 | -0.06 | 1 |  |  |  |  |  |  |  |  |
| 16 | **Sex (reference: women)** |  | -0.03 | 0.12 | 0.04 | -0.10 | -0.05 | 0.03 | -0.02 | -0.03 | 0.02 | -0.03 | 0.08 | 0.01 | -0.03 | 0.02 | 0.06 | 1 |  |  |  |  |  |  |  |
| 17 | **Age at PhD** |  | -0.10 | -0.13 | -0.11 | 0.00 | -0.12 | 0.83 | -0.09 | -0.08 | 0.10 | 0.02 | -0.03 | -0.02 | -0.27 | -0.03 | 0.00 | 0.06 | 1 |  |  |  |  |  |  |
|  | **Research field: Biology and Biomedical Sciences** |  |  |  |  |  |  |  |  |  |  |  |  |  |  |  |  |  |  |  |  |  |  |  |  |
| 18 |  | Exact and Natural Sciences | -0.03 | 0.03 | 0.08 | 0.03 | -0.02 | -0.05 | -0.02 | 0.00 | -0.09 | -0.08 | 0.06 | -0.02 | 0.16 | 0.05 | -0.04 | -0.02 | -0.12 | 1 |  |  |  |  |  |
| 19 |  | Engineering and Technological Sciences | -0.16 | 0.12 | -0.05 | -0.12 | -0.10 | 0.06 | -0.02 | -0.03 | 0.10 | -0.02 | 0.03 | -0.01 | -0.23 | -0.13 | -0.02 | 0.17 | 0.15 | -0.43 | 1 |  |  |  |  |
|  | **Research orientation of the university granting the tenure (reference: high)** |  |  |  |  |  |  |  |  |  |  |  |  |  |  |  |  |  |  |  |  |  |  |  |  |
| 20 |  | Medium | 0.04 | -0.04 | -0.03 | 0.47 | -0.25 | 0.00 | -0.02 | 0.06 | -0.04 | 0.20 | -0.04 | -0.02 | 0.06 | 0.04 | 0.08 | -0.07 | 0.00 | 0.02 | -0.04 | 1 |  |  |  |
| 21 |  | Low | -0.04 | 0.04 | -0.02 | -0.42 | 0.66 | -0.08 | 0.00 | 0.03 | -0.01 | -0.22 | 0.00 | -0.01 | 0.03 | -0.05 | -0.06 | 0.02 | -0.09 | 0.02 | -0.07 | -0.54 | 1 |  |  |
|  | **Labor market (reference: low)** |  |  |  |  |  |  |  |  |  |  |  |  |  |  |  |  |  |  |  |  |  |  |  |  |
| 22 |  | Medium | 0.00 | -0.01 | -0.06 | 0.00 | 0.10 | -0.04 | 0.04 | 0.12 | -0.02 | 0.20 | -0.05 | -0.05 | 0.01 | -0.01 | -0.02 | 0.01 | -0.05 | -0.04 | 0.06 | 0.29 | -0.06 | 1 |  |
| 23 |  | High | -0.10 | 0.07 | -0.01 | -0.17 | 0.13 | 0.01 | -0.07 | -0.11 | 0.05 | -0.46 | 0.08 | 0.03 | -0.07 | -0.01 | -0.03 | 0.08 | 0.04 | 0.10 | 0.13 | -0.22 | 0.25 | -0.41 | 1 |

**Table S4. Time to obtaining a permanent academic job, parametric and non-parametric models**

| **Variable** |  | **Exponential** | | **Weibull** | | **Gompertz** | | **Log-Logistic*** | | **Cox** | |
| --- | --- | --- | --- | --- | --- | --- | --- | --- | --- | --- | --- |
|  |  | **Coeff.** | **P>\|z\|** | **Coeff.** | **P>\|z\|** | **Coeff.** | **P>\|z\|** | **Coeff.** | **P>\|z\|** | **Coeff.** | **P>\|z\|** |
| **Main** |  |  |  |  |  |  |  |  |  |  |  |
| Early publications (ln) |  | -0.001 | 0.990 | 0.656 | 0.000 | 0.587 | 0.000 | **-0.250** | **0.000** | 0.535 | 0.000 |
| Postdoctoral publications by-year (ln) |  | 0.313 | 0.000 | -0.728 | 0.000 | -0.042 | 0.621 | **0.029** | **0.290** | -0.437 | 0.000 |
| Research orientation of the university granting the PhD (reference: High) | Medium | -0.218 | 0.013 | -0.423 | 0.000 | -0.351 | 0.000 | **0.184** | **0.000** | -0.366 | 0.000 |
|  | Low | -0.156 | 0.151 | -0.265 | 0.018 | -0.187 | 0.093 | **0.133** | **0.009** | -0.230 | 0.037 |
| Time to PhD degree |  | -0.022 | 0.181 | -0.031 | 0.050 | -0.039 | 0.009 | **0.013** | **0.118** | -0.030 | 0.064 |
| Involvement in research groups (reference: no) |  | 0.214 | 0.090 | 0.452 | 0.000 | 0.397 | 0.003 | **-0.216** | **0.000** | 0.430 | 0.001 |
| Collaboration with the supervisor (reference: no) |  | 0.176 | 0.019 | 0.344 | 0.000 | 0.339 | 0.000 | **-0.145** | **0.000** | 0.297 | 0.000 |
| University service (reference: no) |  | 0.020 | 0.739 | 0.032 | 0.595 | 0.023 | 0.705 | **-0.013** | **0.627** | 0.042 | 0.482 |
| Inbred status (reference: no) |  | 0.173 | 0.025 | 0.328 | 0.000 | 0.327 | 0.000 | **-0.133** | **0.000** | 0.280 | 0.000 |
| Place of PhD (reference: Spain) |  | -0.108 | 0.637 | -0.040 | 0.861 | 0.022 | 0.924 | **0.058** | **0.587** | -0.057 | 0.804 |
| Postdoctoral international mobility (reference: no) | Yes, short stay (<6 months) | -0.107 | 0.143 | -0.202 | 0.006 | -0.170 | 0.021 | **0.084** | **0.016** | -0.181 | 0.014 |
|  | Yes, long stay | -0.178 | 0.019 | -0.290 | 0.000 | -0.232 | 0.003 | **0.169** | **0.000** | -0.270 | 0.000 |
| Mobility across research groups (reference: no) |  | -0.089 | 0.183 | -0.213 | 0.002 | -0.186 | 0.006 | **0.101** | **0.001** | -0.184 | 0.006 |
| Mobility outside academia (reference: no) |  | -0.284 | 0.083 | -0.668 | 0.000 | -0.889 | 0.000 | **0.286** | **0.000** | -0.520 | 0.002 |
| Sex (reference: women) |  | 0.020 | 0.757 | -0.038 | 0.555 | -0.075 | 0.245 | **-0.041** | **0.163** | -0.008 | 0.896 |
| Age at PhD |  | 0.043 | 0.002 | 0.073 | 0.000 | 0.082 | 0.000 | **-0.040** | **0.000** | 0.063 | 0.000 |
| Research field: (reference: Biology and Biomedical Sciences) | Exact and Natural Sciences | 0.290 | 0.009 | 0.536 | 0.000 | 0.467 | 0.000 | **-0.271** | **0.000** | 0.481 | 0.000 |
|  | Engineering and Technological Sciences | 0.717 | 0.000 | 1.026 | 0.000 | 0.911 | 0.000 | **-0.662** | **0.000** | 0.989 | 0.000 |
| Research field × Postdoctoral Publications (PPUB)-by-year (ln) (reference: Biology and Biomedical Sciences x PPUB) | Exact and Natural Sciences × PPUB | -0.082 | 0.283 | -0.051 | 0.796 | -0.331 | 0.002 | **0.057** | **0.100** | 0.027 | 0.835 |
|  | Engineering and Technological Sciences × PPUB | -0.203 | 0.006 | 0.484 | 0.004 | -0.232 | 0.011 | **0.122** | **0.002** | 0.253 | 0.042 |
| Research orientation of the university granting the tenure (reference: High) | Medium | 0.120 | 0.171 | 0.168 | 0.055 | 0.100 | 0.254 | **-0.064** | **0.123** | 0.177 | 0.044 |
|  | Low | 0.159 | 0.120 | 0.226 | 0.033 | 0.154 | 0.148 | **-0.105** | **0.024** | 0.232 | 0.027 |
| Labor market (reference: Low) | Medium | 0.060 | 0.428 | 0.084 | 0.262 | 0.019 | 0.802 | **-0.081** | **0.022** | 0.104 | 0.170 |
|  | High | 0.207 | 0.009 | 0.391 | 0.000 | 0.315 | 0.000 | **-0.185** | **0.000** | 0.361 | 0.000 |
| Constant |  | -3.821 | 0.000 | -7.167 | 0.000 | -6.095 | 0.000 | **3.437** | **0.000** | - | - |
| **Time-Varying Covariates** |  |  |  |  |  |  |  |  |  |  |  |
| Postdoctoral publications (PPUB) by-year (ln) × time |  | - | - | 0.141 | 0.000 | 0.010 | 0.231 | **-0.168** | **0.000** | 0.078 | 0.000 |
| Research field × Postdoctoral Publications (PPUB) -by-year (ln) (reference: Biology and Biomedical Sciences × PPUB. × time) | Exact and Natural Sciences × PPUB × time | - | - | -0.008 | 0.771 | 0.048 | 0.000 | **0.041** | **0.369** | -0.020 | 0.208 |
|  | Engineering and Technological Sciences × PPUB × time | - | - | -0.166 | 0.000 | -0.012 | 0.310 | **0.180** | **0.000** | -0.097 | 0.000 |
| Constant × time |  | - | - | 0.721 | 0.000 | 0.192 | 0.000 | **-1.198** | **0.000** | - | - |
| Time at risk |  | 7256 |  | 7256 |  | 7256 |  | **7256** |  | 7256 |  |
| Number of subjects |  | 1257 |  | 1257 |  | 1257 |  | **1257** |  | 1257 |  |
| Number of failures |  | 1257 |  | 1257 |  | 1257 |  | **1257** |  | 1257 |  |
| Log Likelihood |  | -1392.5 |  | -927.6 |  | -1069.0 |  | **-898.6** |  | -7622.6 |  |
| LR Chi-square |  | 210.22 |  | 547.01 |  | 464.48 |  | **697.61** |  | 478.85 |  |
| Prob>Chi-square |  | 0.000 |  | 0.000 |  | 0.000 |  | **0.000** |  | 0.000 |  |
| Akaike |  | 2835.0 |  | 1909.2 |  | 2190.5 |  | **1847.7** |  | 15299.2 |  |

**Table S5. Cox semiparametric model with time-varying covariates**

|  | **Variables** |  | **Coeff.** | **S.E.** | **z** | **P>\|z\|** | **Hazard rate** |
| --- | --- | --- | --- | --- | --- | --- | --- |
| **Main** | Early publications (ln) |  | 0.535 | 0.066 | 8.05 | 0.000 | 1.707 |
|  | Postdoctoral publications by-year (ln) |  | -0.437 | 0.118 | -3.70 | 0.000 | 0.646 |
|  | Research orientation of the university granting the PhD (reference: High) | Medium | -0.366 | 0.088 | -4.15 | 0.000 | 0.693 |
|  |  | Low | -0.230 | 0.110 | -2.09 | 0.037 | 0.794 |
|  | Time to PhD degree |  | -0.030 | 0.016 | -1.85 | 0.064 | 0.970 |
|  | Involvement in research groups (reference: no) |  | 0.430 | 0.129 | 3.34 | 0.001 | 1.537 |
|  | Collaboration with the supervisor (reference: no) |  | 0.297 | 0.076 | 3.89 | 0.000 | 1.346 |
|  | University service (reference: no) |  | 0.042 | 0.060 | 0.70 | 0.482 | 1.043 |
|  | Inbred status (reference: no) |  | 0.280 | 0.078 | 3.58 | 0.000 | 1.323 |
|  | Place of PhD (reference: Spain) |  | -0.057 | 0.230 | -0.25 | 0.804 | 0.944 |
|  | Postdoctoral international mobility (reference: no) | Yes, short stay (<6 months) | -0.181 | 0.074 | -2.46 | 0.014 | 0.835 |
|  |  | Yes, long stay | -0.270 | 0.077 | -3.52 | 0.000 | 0.763 |
|  | Mobility across research groups (reference: no) |  | -0.184 | 0.068 | -2.72 | 0.006 | 0.832 |
|  | Mobility outside academia (reference: no) |  | -0.520 | 0.168 | -3.09 | 0.002 | 0.595 |
|  | Sex (reference: women) |  | -0.008 | 0.064 | -0.13 | 0.896 | 0.992 |
|  | Age at PhD |  | 0.063 | 0.013 | 4.85 | 0.000 | 1.065 |
|  | Research field (reference: Biology and Biomedical Sciences) | Exact and Natural Sciences | 0.481 | 0.110 | 4.38 | 0.000 | 1.618 |
|  |  | Engineering and Technological Sciences | 0.989 | 0.110 | 9.03 | 0.000 | 2.690 |
|  | Research field × Postdoctoral Publications (PPUB) by-year (ln) (reference: Biology and Biomedical Sciences × PPUB) | Exact and Natural Sciences × PPUB. | 0.027 | 0.132 | 0.21 | 0.835 | 1.028 |
|  |  | Engineering and Technological Sciences × PPUB | 0.253 | 0.124 | 2.04 | 0.042 | 1.288 |
|  | Research orientation of the university granting the tenure (reference: High) | Medium | 0.177 | 0.088 | 2.01 | 0.044 | 1.193 |
|  |  | Low | 0.232 | 0.105 | 2.21 | 0.027 | 1.261 |
|  | Labor market (reference: Low) | Medium | 0.104 | 0.076 | 1.37 | 0.170 | 1.109 |
|  |  | High | 0.361 | 0.081 | 4.45 | 0.000 | 1.435 |
| **Time-Varying Covariates** | Postdoctoral publications (PPUB) by-year (ln) × time |  | 0.078 | 0.013 | 5.81 | 0.000 | 1.082 |
|  | Research field × Postdoctoral Publications (PPUB) -by-year (ln) × time (reference: Biology and Biomedical Sciences x PPUB × time) | Exact and Natural Sciences × PPUB × time | -0.020 | 0.016 | -1.26 | 0.208 | 0.981 |
|  |  | Engineering and Technological Sciences × PPUB × time | -0.097 | 0.018 | -5.27 | 0.000 | 0.908 |
|  | Time at risk |  | 7256 |  |  |  |  |
|  | Number of subjects |  | 1257 |  |  |  |  |
|  | Number of failures |  | 1257 |  |  |  |  |
|  | Log Likelihood |  | -7622.6 |  |  |  |  |
|  | LR Chi-square |  | 478.85 |  |  |  |  |
|  | Prob>Chi-square |  | 0.000 |  |  |  |  |
|  | **Akaike** |  | **15299** |  |  |  |  |

**Table S6. Cox semiparametric model with time-varying covariate by Research Field**

|  | Variables |  | General | | | Biology and Biomedical Sciences | | | Exact and Natural Sciences | | | Engineering and Technological Sciences | | |
| --- | --- | --- | --- | --- | --- | --- | --- | --- | --- | --- | --- | --- | --- | --- |
|  |  |  | Coeff. | P>\|z\| | Hazard | Coeff. | P>\|z\| | Hazard | Coeff. | P>\|z\| | Hazard | Coeff. | P>\|z\| | Hazard |
| **Main** | Early publications (ln) |  | 0.535 | 0.000 | 1.707 | 0.801 | 0.000 | 2.228 | 0.709 | 0.000 | 2.032 | 0.174 | 0.118 | 1.191 |
|  | Postdoctoral publications (PPUB)-by-year (ln) |  | -0.437 | 0.000 | 0.646 | -0.329 | 0.074 | 0.719 | -0.354 | 0.016 | 0.702 | -0.158 | 0.204 | 0.854 |
|  | Research orientation of the university granting the PhD (reference: high) | Medium | -0.366 | 0.000 | 0.693 | -0.391 | 0.046 | 0.677 | -0.240 | 0.113 | 0.787 | -0.768 | 0.000 | 0.464 |
|  |  | Low | -0.230 | 0.037 | 0.794 | -0.359 | 0.158 | 0.698 | -0.059 | 0.743 | 0.943 | -0.424 | 0.025 | 0.654 |
|  | Time to PhD degree |  | -0.030 | 0.064 | 0.970 | -0.072 | 0.110 | 0.931 | -0.002 | 0.938 | 0.998 | -0.032 | 0.205 | 0.968 |
|  | Involvement in research groups (reference: no) |  | 0.430 | 0.001 | 1.537 | 0.213 | 0.368 | 1.237 | 0.472 | 0.039 | 1.604 | 0.515 | 0.021 | 1.674 |
|  | Collaboration with the supervisor (reference: no) |  | 0.297 | 0.000 | 1.346 | 0.306 | 0.032 | 1.357 | 0.193 | 0.132 | 1.213 | 0.286 | 0.034 | 1.331 |
|  | University service (reference: no) |  | 0.042 | 0.482 | 1.043 | 0.176 | 0.111 | 1.193 | -0.052 | 0.611 | 0.949 | -0.021 | 0.852 | 0.980 |
|  | Inbred status (reference: no) |  | 0.280 | 0.000 | 1.323 | 0.156 | 0.292 | 1.168 | 0.206 | 0.122 | 1.229 | 0.470 | 0.001 | 1.599 |
|  | Place of PhD (reference: Spain) |  | -0.057 | 0.804 | 0.944 | -0.613 | 0.419 | 0.542 | 0.119 | 0.719 | 1.126 | -0.098 | 0.799 | 0.907 |
|  | Postdoctoral international mobility (reference: no) | Yes, short stay (<6 months) | -0.181 | 0.014 | 0.835 | -0.128 | 0.356 | 0.880 | -0.137 | 0.293 | 0.872 | -0.118 | 0.354 | 0.889 |
|  |  | Yes, long stay | -0.270 | 0.000 | 0.763 | -0.073 | 0.583 | 0.930 | -0.375 | 0.003 | 0.687 | -0.354 | 0.041 | 0.702 |
|  | Mobility across research groups (reference: no) |  | -0.184 | 0.006 | 0.832 | -0.153 | 0.204 | 0.858 | -0.154 | 0.169 | 0.857 | -0.282 | 0.042 | 0.754 |
|  | Mobility outside academia (reference: no) |  | -0.520 | 0.002 | 0.595 | -0.300 | 0.260 | 0.741 | -0.878 | 0.008 | 0.416 | -0.475 | 0.159 | 0.622 |
|  | Sex (reference: women) |  | -0.008 | 0.896 | 0.992 | -0.146 | 0.188 | 0.864 | 0.192 | 0.064 | 1.212 | -0.181 | 0.169 | 0.835 |
|  | Age at PhD |  | 0.063 | 0.000 | 1.065 | 0.086 | 0.030 | 1.090 | 0.069 | 0.002 | 1.072 | 0.067 | 0.001 | 1.069 |
|  | Research field (reference: Biology and Biomedical Sciences) | Exact and Natural Sciences | 0.481 | 0.000 | 1.618 | - | - | - | - | - | - | - | - | - |
|  |  | Engineering and Technological Sciences | 0.989 | 0.000 | 2.690 | - | - | - | - | - | - | - | - | - |
|  | Research field × Postdoctoral Publications (PPUB) year-by-year (ln) (reference: Biology and Biomedical Sciences x PPUB) | Exact and Natural Sciences × PPUB. | 0.027 | 0.835 | 1.028 | - | - | - | - | - | - | - | - | - |
|  |  | Engineering and Technological Sciences × PPUB | 0.253 | 0.042 | 1.288 | - | - | - | - | - | - | - | - | - |
|  | Research orientation of the university granting the tenure (reference: High) | Medium | 0.177 | 0.044 | 1.193 | 0.168 | 0.313 | 1.183 | -0.002 | 0.987 | 0.998 | 0.574 | 0.001 | 1.776 |
|  |  | Low | 0.232 | 0.027 | 1.261 | 0.214 | 0.323 | 1.239 | 0.007 | 0.969 | 1.007 | 0.528 | 0.004 | 1.695 |
|  | Labor market (reference: Low) | Medium | 0.104 | 0.170 | 1.109 | 0.149 | 0.310 | 1.161 | 0.204 | 0.108 | 1.226 | -0.154 | 0.278 | 0.857 |
|  |  | High | 0.361 | 0.000 | 1.435 | 0.539 | 0.003 | 1.714 | 0.300 | 0.025 | 1.350 | 0.233 | 0.101 | 1.262 |
| **Time-Varying Covariates** | Postdoctoral publications (PPUB) by-year (ln) × time |  | 0.078 | 0.000 | 1.082 | 0.048 | 0.020 | 1.049 | 0.036 | 0.065 | 1.036 | 0.040 | 0.029 | 1.041 |
|  | Research field × Postdoctoral Publications (PPUB) -by-year (ln) × time (reference: Biology and Biomedical Sciences x PPUB × time) | Exact and Natural Sciences × PPUB × time | -0.020 | 0.208 | 0.981 | - | - | - | - | - | - | - | - | - |
|  |  | Engineering and Technological Sciences × PPUB × time | -0.097 | 0.000 | 0.908 | - | - | - | - | - | - | - | - | - |
|  | Time at risk |  | 7256 |  |  | 2835 |  |  | 2786 |  |  | 1635 |  |  |
|  | Number of subjects |  | 1257 |  |  | 371 |  |  | 463 |  |  | 423 |  |  |
|  | Number of failures |  | 1257 |  |  | 371 |  |  | 463 |  |  | 423 |  |  |
|  | Log Likelihood |  | -7623 |  |  | -1825 |  |  | -2384 |  |  | -2161 |  |  |
|  | LR Chi-square |  | 478.85 |  |  | 94.18 |  |  | 116.49 |  |  | 87.33 |  |  |
|  | Prob>Chi-square |  | 0.000 |  |  | 0.000 |  |  | 0.000 |  |  | 0.000 |  |  |
|  | **Akaike** |  | **15299** |  |  | **3692** |  |  | **4810** |  |  | **4364** |  |  |

**Table S7. Cox semiparametric model with time-varying covariate by type of international mobility**

|  | Variables |  | General | | | Postdoctoral international mobility: no | | | Postdoctoral international mobility: Yes, short stay (<6 months) | | | Postdoctoral international mobility: Yes, long stay. | | |
| --- | --- | --- | --- | --- | --- | --- | --- | --- | --- | --- | --- | --- | --- | --- |
|  |  |  | Coeff. | P>\|z\| | Hazard | Coeff. | P>\|z\| | Hazard | Coeff. | P>\|z\| | Hazard | Coeff. | P>\|z\| | Hazard |
| **Main** | Early publications (ln) |  | 0.535 | 0.000 | 1.707 | 0.373 | 0.000 | 1.452 | 0.483 | 0.000 | 1.620 | 0.868 | 0.000 | 2.381 |
|  | Postdoctoral publications (PPUB)-by-year (ln) |  | -0.437 | 0.000 | 0.646 | -0.360 | 0.041 | 0.698 | -0.605 | 0.022 | 0.546 | -0.202 | 0.408 | 0.817 |
|  | Research orientation of the university granting the PhD (reference: High) | Medium | -0.366 | 0.000 | 0.693 | -0.495 | 0.000 | 0.610 | -0.273 | 0.140 | 0.761 | -0.149 | 0.402 | 0.861 |
|  |  | Low | -0.230 | 0.037 | 0.794 | -0.318 | 0.048 | 0.728 | -0.100 | 0.660 | 0.905 | -0.017 | 0.942 | 0.984 |
|  | Time to PhD degree |  | -0.030 | 0.064 | 0.970 | -0.048 | 0.031 | 0.953 | -0.010 | 0.784 | 0.990 | 0.079 | 0.125 | 1.082 |
|  | Involvement in research groups (reference: no) |  | 0.430 | 0.001 | 1.537 | 0.491 | 0.007 | 1.634 | 0.080 | 0.811 | 1.083 | 0.519 | 0.043 | 1.680 |
|  | Collaboration with the supervisor (reference: no) |  | 0.297 | 0.000 | 1.346 | 0.431 | 0.000 | 1.538 | 0.075 | 0.674 | 1.077 | 0.246 | 0.072 | 1.279 |
|  | University service (reference: no) |  | 0.042 | 0.482 | 1.043 | -0.013 | 0.886 | 0.987 | 0.225 | 0.092 | 1.252 | -0.039 | 0.741 | 0.962 |
|  | Inbred status (reference: no) |  | 0.280 | 0.000 | 1.323 | 0.274 | 0.020 | 1.315 | 0.564 | 0.000 | 1.757 | 0.043 | 0.788 | 1.044 |
|  | Place of PhD (reference: Spain) |  | -0.057 | 0.804 | 0.944 | 0.104 | 0.786 | 1.109 | 0.457 | 0.390 | 1.580 | -0.431 | 0.281 | 0.650 |
|  | Postdoctoral international mobility (reference: no) | Yes, short stay (<6 months) | -0.181 | 0.014 | 0.835 | - | - | - | - | - | - | - | - | - |
|  |  | Yes, long stay | -0.270 | 0.000 | 0.763 | - | - | - | - | - | - | - | - | - |
|  | Mobility across research groups (reference: no) |  | -0.184 | 0.006 | 0.832 | -0.150 | 0.168 | 0.861 | -0.236 | 0.105 | 0.789 | -0.318 | 0.011 | 0.727 |
|  | Mobility outside academia (reference: no) |  | -0.520 | 0.002 | 0.595 | -0.324 | 0.140 | 0.723 | -0.664 | 0.082 | 0.515 | -1.066 | 0.009 | 0.344 |
|  | Sex (reference: women) |  | -0.008 | 0.896 | 0.992 | -0.114 | 0.248 | 0.893 | 0.182 | 0.212 | 1.199 | 0.018 | 0.882 | 1.018 |
|  | Age at PhD |  | 0.063 | 0.000 | 1.065 | 0.079 | 0.000 | 1.082 | 0.052 | 0.054 | 1.054 | 0.018 | 0.627 | 1.018 |
|  | Research field (reference: Biology and Biomedical Sciences) | Exact and Natural Sciences | 0.481 | 0.000 | 1.618 | 0.570 | 0.001 | 1.768 | 0.693 | 0.004 | 1.999 | 0.346 | 0.078 | 1.413 |
|  |  | Engineering and Technological Sciences | 0.989 | 0.000 | 2.690 | 1.011 | 0.000 | 2.748 | 1.022 | 0.000 | 2.778 | 0.929 | 0.002 | 2.532 |
|  | Research field × Postdoctoral Publications (PPUB) by-year (ln) (reference: Biology and Biomedical Sciences x PPUB) | Exact and Natural Sciences × PPUB | 0.027 | 0.835 | 1.028 | 0.102 | 0.611 | 1.107 | 0.105 | 0.703 | 1.111 | -0.023 | 0.933 | 0.978 |
|  |  | Engineering and Technological Sciences × PPUB | 0.253 | 0.042 | 1.288 | 0.343 | 0.048 | 1.410 | 0.305 | 0.260 | 1.357 | 0.256 | 0.474 | 1.292 |
|  | Research orientation of the university granting the tenure (reference: High) | Medium | 0.177 | 0.044 | 1.193 | 0.342 | 0.015 | 1.408 | 0.196 | 0.262 | 1.216 | -0.190 | 0.288 | 0.827 |
|  |  | Low | 0.232 | 0.027 | 1.261 | 0.279 | 0.066 | 1.322 | 0.426 | 0.048 | 1.532 | -0.203 | 0.346 | 0.816 |
|  | Labor market (reference: Low) | Medium | 0.104 | 0.170 | 1.109 | 0.113 | 0.311 | 1.119 | 0.170 | 0.332 | 1.185 | 0.207 | 0.166 | 1.230 |
|  |  | High | 0.361 | 0.000 | 1.435 | 0.406 | 0.001 | 1.501 | 0.384 | 0.022 | 1.468 | 0.304 | 0.052 | 1.356 |
| **Time-Varying Covariates** | Postdoctoral publications (PPUB) by-year (ln) × time |  | 0.078 | 0.000 | 1.082 | 0.086 | 0.000 | 1.090 | 0.095 | 0.001 | 1.099 | 0.031 | 0.258 | 1.031 |
|  | Research field × Postdoctoral Publications (PPUB) -by-year (ln) × time (reference: Biology and Biomedical Sciences x PPUB × time) | Exact and Natural Sciences × PPUB × time | -0.020 | 0.208 | 0.981 | -0.039 | 0.112 | 0.962 | -0.028 | 0.404 | 0.973 | -0.010 | 0.749 | 0.990 |
|  |  | Engineering and Technological Sciences × PPUB × time | -0.097 | 0.000 | 0.908 | -0.118 | 0.000 | 0.889 | -0.097 | 0.007 | 0.907 | -0.099 | 0.095 | 0.906 |
|  | Time at risk |  | 7256 |  |  | 2973 |  |  | 1727 |  |  | 2556 |  |  |
|  | Number of subjects |  | 1257 |  |  | 600 |  |  | 296 |  |  | 361 |  |  |
|  | Number of failures |  | 1257 |  |  | 600 |  |  | 296 |  |  | 361 |  |  |
|  | Log Likelihood |  | -7623 |  |  | -3207 |  |  | -1364 |  |  | -1758 |  |  |
|  | LR Chi-square |  | 478.9 |  |  | 215.84 |  |  | 119.66 |  |  | 117.17 |  |  |
|  | Prob>Chi-square |  | 0.000 |  |  | 0.000 |  |  | 0.000 |  |  | 0.000 |  |  |
|  | **Akaike** |  | **15299** |  |  | **6464** |  |  | **2779** |  |  | **3565** |  |  |
