# Supplementary material for: Time to Tenure in Spanish Universities: An Event History Analysis
Source: PLoS One. 2013 Oct 8;8(10):e77028. doi: 10.1371/journal.pone.0077028 (PMC3792917; doi:10.1371/journal.pone.0077028)
Supplement: File S2 — Questionnaire. (PDF) [file pone.0077028.s002.pdf]

Cuestionario sobre Trayectorias Profesionales a Profesores de Universidad e Investigadores del CSIC

Grupo de Investigación sobre Políticas de Innovación, Tecnología, Formación y Educación **(SPRITTE)**

CONSEJO SUPERIOR
DE INVESTIGACIONES
CIENTÍFICAS

UNIDAD DE POLÍTICAS COMPARADAS

Cuestionario sobre Trayectorias Profesionales a Profesores de Universidad e Investigadores del CSIC

Grupo de **I**nvestigación sobre **P**olíticas
de **I**nnovación, **T**ecnología, **F**ormación
y **E**ducación **(SPRITTE)**

Por favor, responda a todas las preguntas.

Use el espacio de la última página si desea escribir cualquier comentario adicional.

Cuando finalice el cuestionario, introdúzcalo en el sobre adjunto y deposítelo en el correo.

Muchas gracias por su colaboración

El cuestionario que presentamos a continuación forma parte de un proyecto de investigación sobre los recursos humanos en la docencia y la investigación en España y, más específicamente, sobre las carreras académicas e investigadoras. Este proyecto es llevado a cabo por el grupo de investigación SPRITTE (Spanish Policy Research on Innovation & Technology, Training & Education), *de la Unidad de Políticas Comparadas del CSIC, y está dirigido por Laura Cruz Castro y financiado por el Plan Nacional de I+D+I, 2000-2003 (SEC-2001-2411-C02-01).*

El objetivo de este cuestionario es recoger información acerca de la experiencia profesional e investigadora de aquellos ***Profesores Titulares de Universidad y Científicos Titulares del CSIC que accedieron a una plaza entre el 1 de enero de 1997 y 31 de diciembre de 2001.*** *Con dicho fin, las preguntas están organizadas en bloques temáticos, abarcando cada una de las etapas dentro de la carrera docente-investigadora.*

Sus respuestas serán tratadas en todo momento de manera confidencial. Cualquier información que se elabore y/o publique sólo se hará en forma agregada y debidamente anonimizada. Asimismo, el tratamiento de la información se hace al amparo de las correspondientes Leyes sobre secreto estadístico y protección de datos personales.

MUCHAS GRACIAS POR SU COLABORACIÓN

Este cuestionario tiene un tiempo aproximado de realización de entre 15 y 20 min. Por favor, antes de comenzar, lea atentamente las instrucciones.

Instrucciones para rellenar el cuestionario

- *Rodee con un círculo el número correspondiente a la opción elegida en cada pregunta. Por favor, CONTESTE A TODAS LAS PREGUNTAS.*
- *Escriba en MAYÚSCULAS sus respuestas en las preguntas abiertas.*
- *Si se equivoca, tache con una cruz el círculo incorrecto y vuelva a rodear la opción elegida.*
- *Marque sólo una opción salvo en aquellas preguntas en las que se indique previamente la posibilidad de marcar dos o más opciones.*
- *En algunas preguntas se añade la opción «Otro», seguida de una línea para rellenar. Escriba en esta línea lo que considere más adecuado, si entre las opciones indicadas no encuentra ninguna apropiada para responder de acuerdo con su opinión.*
- *Las preguntas incluidas en recuadros sombreados son «preguntas filtro». Sólo pueden contestarse si previamente se ha respondido a la opción correspondiente que se indica en una pregunta anterior.*
- *Algunas preguntas requieren que se rellenen cuadros o tablas. En estos casos, por favor, siga las instrucciones específicas que se indican en estas preguntas.*
- *Finalmente, dado que parte de la información que se solicita hace referencia a la carrera docente-investigadora del entrevistado, se recomienda que éste, a la hora de rellenarlo, TENGA A MANO UN CURRÍCULUM VITAE PARA FACILITAR LA CONTESTACIÓN A LAS PREGUNTAS.*

Para cualquier duda a la hora de cumplimentar el cuestionario, no dude en ponerse en contacto con:

Grupo de Investigación sobre Políticas de Innovación,
Tecnología, Formación y Educación (Grupo SPRITTE)
Unidad de Políticas Comparadas – CSIC
C/ Alfonso XII, 18, 5.º, 28014 Madrid
TEL: 91 521 90 28 (extensiones 119, 118, 105 y 110)
FAX: 91 521 81 03 e-mail: jaime.aja@iesam.csic.es o jose.real@iesam.csic.es
www.iesam.csic.es/spritte.htm

1 DATOS PERSONALES

1. **Para comenzar, necesitamos que complete el siguiente cuadro con algunos datos sobre su nacimiento y nacionalidad:**

**Fecha de nacimiento** (día, mes y año)		
**Lugar de nacimiento**	Provincia:	
	País:	
**Nacionalidad**		

2. **Rellene la siguiente tabla con los datos sobre su *puesto académico actual:***

**Puesto académico actual** (rodee con un círculo el código que corresponda)			**Fecha de toma de posesión** (mes y año)
	Profesor Titular de Universidad	1	
	Científico Titular del CSIC	2	
	Catedrático de Universidad	3	
	Investigador Científico del CSIC	4	
	Profesor de Investigación del CSIC	5	
**Área de conocimiento**			
**Departamento / Centro**			
**Universidad * / CSIC** *(nombre completo o abreviatura)			

**Dedicación** (rodee con un círculo el código que corresponda)	Tiempo parcial	1	Tiempo completo	2

3. **¿Ha ocupado con anterioridad al puesto actual otra plaza de Profesor Titular de Universidad o de Científico Titular del CSIC?**

Sí	1	➔	Pase a la pregunta 4
No	2	➔	Pase a la pregunta 5

SI HA OCUPADO OTROS PUESTOS DE PROFESOR TITULAR O CIENTÍFICO TITULAR CON ANTERIORIDAD

4. **Por favor, señale la universidad o centro, el área de conocimiento y la fecha de obtención de los puestos obtenidos con anterioridad al actual:**

	Área de conocimiento	Universidad o Centro del CSIC (nombre completo o abreviatura)	Fecha de obtención (mes y año)
Profesor titular			
Científico titular			

PASE A LA PREGUNTA 5

5. **¿Qué estudios tenía su padre (estudios finalizados)? ¿Y su madre?**

**Estudios finalizados** (rodee con un círculo donde corresponda)	**Padre**	**Madre**
Sin estudios	1	1
Primarios	2	2
Secundarios (Bachillerato, FP)	3	3
Universitarios	4	4
Doctorado	5	5

6. **¿Tiene o ha tenido familiares en el mundo universitario-investigador? Rodee con un círculo las respuestas que considere pertinentes.**

No	1
Sí, mi padre y/o mi madre	2
Sí, hermano/s y/o hermana/s	3
Sí, otros parientes en primer grado	4

7. **Por favor, en la siguiente tabla, señale los títulos universitarios y de doctorado que posee.**

	**Nombre de la titulación**	**Universidad que la ortorgó** (nombre completo o abreviatura)	**País**	**Año de obtención**
**Licenciado, Ingeniero o equivalente en**				
**Doctor en**				

2 SITUACIÓN ACTUAL

Las preguntas que aparecen dentro de esta sección están referidas a diferentes aspectos relacionados con su carrera profesional desde que accedió a un puesto de Profesor Titular de Universidad o Científico Titular del CSIC por primera vez hasta el momento presente.

8. **Por favor, señale en el siguiente cuadro el tiempo (años y meses) en que ha permanecido en alguna de las siguientes *posiciones profesionales, incluyendo estancias fuera de su centro, después de la obtención de la plaza de Profesor Titular de Universidad o Científico Titular* (por primera vez, en caso de haber obtenido más de una plaza).**

**Posiciones profesionales**		**Tiempo** (en años y meses)
Profesor Titular de Universidad	1	
Científico Titular del CSIC	2	
Catedrático de Universidad	3	
Investigador Científico del CSIC	4	
Profesor de Investigación del CSIC	5	
Posición docente/investigadora permanente en el extranjero	6	
Posición docente/investigadora temporal en el extranjero *(visiting scholar, visiting professor, etc.)*	7	
Trabajo de investigación en el sector privado	8	
Cargo académico (Director Dpto., Decano, Vicerrector, etc.)	9	
Cargo político-administrativo	10	
Otra situación (sabático, excedencia, etc.)	11	

9. **Actualmente, ¿en qué condiciones le puede resultar más atractiva una oferta de trabajo en el sector privado? Por favor, de entre las respuestas que se muestran a continuación rodee con un círculo *la principal:***

Una oferta que sea económicamente interesante	1
Una oferta interesante desde el punto de vista investigador	2
Si me permitiese conciliar mi actividad en la universidad o en el CSIC con el trabajo en el sector privado	3
Si me permitiese retornar a mi puesto en la universidad o en el CSIC en cualquier momento	4
No aceptaría una oferta en el sector privado	5

3 ACCESO AL PUESTO DE PROFESOR TITULAR

En esta sección las preguntas se refieren al proceso de acceso al puesto de Profesor Titular de Universidad o Científico Titular del CSIC. En caso de haber obtenido más de una plaza de titular, tenga en cuenta que las preguntas se refieren a la *primera plaza* que obtuvo.

10. **¿Podría señalar cuántas veces concursó a una plaza de *Profesor Titular de Universidad* con anterioridad a la que finalmente obtuvo? ¿Y cuántas veces concursó a una plaza de Científico Titular del CSIC con anterioridad?**

Plaza	Número de veces que concursó	
Profesor Titular de Universidad		➔ Pase a la pregunta 11
Científico Titular del CSIC		➔ Pase a la pregunta 12

SI CONCURSÓ CON ANTERIORIDAD A ALGUNA PLAZA DE PROFESOR TITULAR EN LA UNIVERSIDAD
(Pregunta 10)

11. **De ellas, ¿cuántas en la universidad donde obtuvo la plaza (exceptuando la que obtuvo finalmente)?**

Número	

PASE A LA PREGUNTA 12

12. **En la oposición en que usted obtuvo la plaza, además de usted, ¿cuántos otros candidatos concurrieron a la plaza?** (Señale *solamente aquéllos que se presentaron de hecho a la plaza, no los que firmaron).*

Número	

13. **¿En cuáles de las siguientes circunstancias ha estado en la universidad o centro del CSIC, donde obtuvo su plaza de titular?** Rodee con un círculo *todas las respuestas que sean necesarias.*

Realicé la carrera en esta universidad	1
Fui becario predoctoral	2
Tuve un contrato antes de ser doctor	3
Realicé una estancia antes de ser doctor	4
Fui becario postdoctoral	5
Tuve un contrato después de ser doctor	6
Realicé una estancia después de ser doctor	7
Había concursado antes a una plaza por esta institución	8
Ninguna de las anteriores	9

14. **¿Trabajaba en esta universidad o centro del CSIC en el momento de conseguir la plaza?**

Sí	1	➔ Pase a la pregunta 15
No, pero había trabajado anteriormente	2	➔ Pase a la pregunta 17
No, no había trabajado nunca	3	➔ Pase a la pregunta 16

SI TRABAJABA EN LA UNIVERSIDAD O CSIC EN EL MOMENTO DE CONSEGUIR LA PLAZA
(«Sí» en la pregunta 14)

15. **¿Cuánto tiempo llevaba trabajando de forma continuada en esta universidad o centro?**

Años	

PASE A LA PREGUNTA 17

SI ANTES NO HABÍA TRABAJADO NUNCA EN LA UNIVERSIDAD O CENTRO DONDE CONSIGUIÓ LA PLAZA
(«No, no había trabajado nunca» en la pregunta 14)

16. **¿Había colaborado anteriormente con profesores o investigadores de esta universidad? Por favor, señale todas las respuestas que correspondan.**

No había colaborado anteriormente	1
Sí, en proyectos de investigación	2
Sí, en artículos u otras publicaciones	3
Sí, en la organización de actividades científicas	4

PASE A LA PREGUNTA 17

17. **¿Ha participado en algún tribunal de oposición o habilitación?**

Sí	1
No	2

18. **En su opinión, en su área científica, de los aspectos que se le ofrecen a continuación, ¿cuáles son *los que más se valoran* a la hora de acceder a una plaza de Profesor Titular o Científico Titular del CSIC? ¿Y cuáles, según su opinión, *cree que deberían valorarse?***
(Señale con una X en cada caso los *tres principales por orden de importancia)*

**Aspecto**	**Se valoran en la práctica** (Orden de valoración)			**Deberían valorarse** (Orden de valoración)		
	**1º**	**2º**	**3º**	**1º**	**2º**	**3º**
Calificación del doctorado y calidad de la Tesis						
Experiencia docente						
Dirección de proyectos de investigación						
Participación en proyectos de investigación						
Publicaciones						
Estancias en centros extranjeros						
Colaboraciones con el sector privado						
Sintonía con las líneas de investigación del departamento						
Antigüedad y permanencia anterior en el departamento						
Apoyo del departamento receptor al candidato						
Otro, ¿cuál?						

4 ETAPA POSTDOCTORAL

Ahora vamos a pedirle que recuerde su experiencia profesional durante la *etapa postdoctoral,* desde el momento en que obtuvo el *grado de doctor* hasta que obtuvo una *plaza de Profesor o Científico Titular.*

19. **Por favor, señale en el siguiente cuadro las *situaciones profesionales por las que pasó desde que leyó la tesis hasta la obtención de la plaza de Profesor Titular en la Universidad o de Científico Titular en el CSIC* (y en las que permaneciese un *mínimo de 4 meses*). *Ordénelas cronológicamente,* utilizando los *códigos* que se acompañan. Señale la institución donde estuvo, el lugar, así como el tiempo que pasó en cada posición, en años y meses.**

Códigos	Situaciones profesionales
1	Becario postdoctoral (salvo los de reincorporación)
2	Becario de reincorporación
3	Contrato postdoctoral (salvo los de reincorporación)
4	Contrato de reincorporación
5	Posición de investigador en el extranjero (research fellow, etc.)
6	Profesor Asociado (tiempo completo)
7	Profesor Asociado (tiempo parcial)
8	Profesor Ayudante
9	Profesor Titular de Escuela Universitaria
10	Profesor Titular interino
11	Científico Titular interino
12	Profesor Universidad Privada
13	Investigador funcionario en Centro Público de Investigación
14	Investigador sin vinculación formal con el centro / honorario / sin contrato
15	En paro/inscrito en el INEM/ Cobrando el subsidio de desempleo/Otros subsidios
16	Inactivo/Sin ingresos/Dependiente ayuda familiar
17	Trabajo fuera del ámbito académico
18	Otros, ¿cuáles?

**Situación** (Introduzca código)	**Institución Universidad/Organismo**	**Lugar** (ciudad y país)	**Tiempo que estuvo en esa situación** (en años y meses)

SI TUVO ALGUNA BECA EN LA ETAPA POSTDOCTORAL (CÓDIGOS 1 Ó 2) CONTESTE A LA PREGUNTA 20
SI TRABAJÓ FUERA DEL ÁMBITO ACADÉMICO EN LA ETAPA POSTDOCTORAL (CÓDIGO 16) CONTESTE A LA PREGUNTA 21
SI NO ESTUVO EN NINGUNA DE ESTAS DOS SITUACIONES (CÓDIGOS 1, 2 Ó 16) PASE DIRECTAMENTE A LA PREGUNTA 22

SI TUVO UNA BECA POST-DOCTORAL
(alguna respuesta con código 1 ó 2 en la pregunta 19)

20. ¿Qué tipo de beca, de más de cuatro meses, obtuvo? Señale el organismo pagador, utilizando los *códigos* siguientes. Señale si la beca estaba vinculada a algún proyecto y si entre sus tareas se encontraba la docencia.

Códigos	Organismo pagador
1	Ministerio de Educación y Ciencia
2	Otros Ministerios
3	Comunidad Autónoma
4	Centro Público de Investigación
5	Universidad
6	Institución privada
7	Organismo internacional
8	Otras, ¿cuáles?

Orden de las becas	Organismo pagador (inserte código)	Vinculado a Proyecto de investigación (rodee con un círculo)	Docencia (rodee con un círculo)
Primera beca		Sí / No	Sí / No
Segunda beca		Sí / No	Sí / No
Tercera beca		Sí / No	Sí / No
Cuarta beca		Sí / No	Sí / No

Si tuvo más becas indíquelo a continuación:

SI TRABAJÓ FUERA DEL ÁMBITO ACADÉMICO EN LA ETAPA POSTDOCTORAL
(CÓDIGO 16 EN LA PREGUNTA 19) RECUERDE CONTESTAR A LA PREGUNTA 21

SI NO PASE DIRECTAMENTE A LA PREGUNTA 22

SI TUVO UN TRABAJO FUERA DEL ÁMBITO ACADÉMICO EN SU ETAPA POST-DOCTORAL
(alguna respuesta con código 16 en la pregunta 19)

21. **¿Qué tipo de trabajo realizó? Marque el tipo de trabajo utilizando los códigos siguientes. Señale también si la dedicación era completa o no y si el trabajo en líneas generales era de investigación o no.**

Códigos	Trabajo fuera del ámbito académico
1	Contrato o colaboración en el sector privado
2	Contrato o colaboración en el sector público
3	Profesor de enseñanzas medias
4	Técnico de la Administración Civil u otros puestos de funcionario del grupo A
5	Empresario
6	Autónomo / Profesión liberal
7	Otros, ¿cuáles?

Orden de los trabajos	Trabajo fuera del ámbito académico (inserte código)	Trabajo de I+D	Dedicación (rodee con un círculo)	
Primer trabajo		Sí	Parcial	1
		No	Completa	2
Segundo trabajo		Sí	Parcial	1
		No	Completa	2
Tercer trabajo		Sí	Parcial	1
		No	Completa	2
Cuarto trabajo		Sí	Parcial	1
		No	Completa	2

PASE A LA PREGUNTA 22

22. **¿Realizó alguna estancia breve** ***(de más de un mes y menos de seis meses)*** **en algún otro centro distinto a aquel al que estaba formalmente vinculado durante el período postdoctoral?**

Sí	1	➔ Pase a la pregunta 23
No	2	➔ Pase a la pregunta 24

SI REALIZÓ ALGUNA ESTANCIA TEMPORAL EN LA ETAPA POSTDOCTORAL
(respuesta 1 en la pregunta 22)

23. Por favor, señale dónde (centro, localidad y país), año y la duración aproximada en meses.

**Centro** (Universidad / Institución)	**Localidad y país**	**Fecha** (año)	**Duración** (en meses)

PASE A LA PREGUNTA 24

24. Durante *el año que siguió a la lectura de la tesis,* por favor, conteste si se produjeron o no los siguientes cambios en su carrera:

	Sí	No
Aumentó mi salario	1	2
Se incrementó mi carga docente	1	2
Mejoró mi estabilidad laboral	1	2

25. ¿Colaboró en trabajos de investigación con su director de tesis después de leerla?

Sí	1
No	2

26. En el año que siguió a la lectura de la tesis doctoral, ¿cambió de universidad/centro público de investigación respecto al que se encontraba en el momento de presentar la tesis?

Sí, cambié de centro	1	➔ Pase a la pregunta 27
No cambié	2	➔ Pase a la pregunta 30

SI CAMBIÓ DE CENTRO O UNIVERSIDAD RESPECTO AL QUE SE ENCONTRABA EN EL MOMENTO DE PRESENTAR LA TESIS
(«Sí, cambié de centro» en la pregunta 26)

27. ¿Existía algún tipo de vínculo entre el centro o grupo de investigación en el que realizó la tesis y la nueva institución?

Sí	1
No	2

28. Por favor, de los siguientes factores, señale los dos que más influyeron en que usted cambiase de centro (rodee con un círculo):

La falta de oportunidades laborales en el centro de origen	1
Avanzar profesionalmente en mi carrera	2
En el centro de origen se exige que se realicen esas estancias	3
Mejores condiciones de trabajo	4
Factores personales y familiares	5
No podía permanecer en el mismo centro después de presentar la tesis	6
Otro, ¿cuál?	7

29. ¿Cuáles fueron los dos factores principales que tuvo en cuenta a la hora de elegir el centro al que se incorporó?

Disponía de financiación para ir a ese centro	1
Me fue recomendado por mi director de tesis	2
El centro mantenía relaciones de colaboración con mi centro de origen	3
El nuevo centro constituía el lugar más adecuado para continuar con mi labor investigadora	4
Había una posición vacante, y concursé	5
Otro, ¿cuál?	6

PASE A LA PREGUNTA 30

30. **¿Perteneció usted de forma continuada a algún grupo de investigación durante la etapa postdoctoral?**

Sí, a un solo grupo	1
Sí, a varios grupos	2
No	3

31. **De la siguiente lista de actividades distintas de la docencia/investigación, señale todas aquéllas en las que participó durante la etapa postdoctoral:**

Actividad	Participó
Participación institucional (claustro, junta de facultad, junta de personal)	1
Asociaciones universitarias de profesorado	2
Asociaciones científicas	3
Afiliación a sindicato	4
Militancia en partido político	5
Cargo de gestión (Secretario de Departamento, Vicedecano, etc.)	6
No participó en ninguna de las anteriores actividades	7

5 ETAPA PREDOCTORAL

En esta sección las preguntas se centran en su *experiencia predoctoral,* desde el momento en que obtuvo el título de Licenciado o Ingeniero hasta que obtuvo el grado de Doctor.

32. **¿En qué año se inscribió por vez primera en los cursos de doctorado?** ....

33. **¿Cuánto *tiempo efectivo* dedicó a la realización de la tesis (n.º años)?**

Años	

34. **¿Cuál fue el factor principal que le impulsó a comenzar el doctorado? Por favor, señale de los siguientes el más importante:**

Completar mi formación universitaria	1
Iniciar la carrera académica	2
Continuar en la carrera académica	3
Situación de desempleo	4
Influencia familiar o del entorno	5
Quería probar la investigación	6
Otro, ¿cuál?	7

35. **Por favor, señale en el siguiente cuadro las** ***situaciones profesionales por las que pasó desde que obtuvo el grado de Licenciado o Ingeniero hasta que obtuvo el grado de doctor*** **por primera vez (y en las que permaneciese un** ***mínimo de 4 meses).*** **Señale el tiempo, en años y meses, que pasó en cada posición. Si pasó por la misma posición varias veces, sume el tiempo.**

**Posición profesional**	(Rodee con un círculo)	**Tiempo que estuvo en esa situación** (en años y meses)
Becario de investigación	1	
Contrato de investigación con cargo a proyecto	2	
Otra posición de investigador en el extranjero *(research fellow,* etc.)	3	
Profesor Asociado (tiempo completo)	4	
Profesor Asociado (tiempo parcial)	5	
Profesor Ayudante	6	
Profesor Titular de Escuela Universitaria	7	
Profesor en Universidad Privada	8	
Investigador sin vinculación formal con el centro	9	
En paro/inscrito en el INEM/ Cobrando el subsidio de desempleo	10	
Inactivo/Sin ingresos/Dependiente ayuda familiar	11	
Trabajo fuera del ámbito académico	12	

SI TUVO UNA BECA PREDOCTORAL (CÓDIGO 1) CONTESTE LA PREGUNTA 36.
SI NO, PASE A LA PREGUNTA 37

SI TUVO UNA BECA PREDOCTORAL
(respuesta 1 en la pregunta 35)

36. Rellene el siguiente cuadro sobre las becas predoctorales de las que disfrutó, indicando el código del organismo pagador de la beca. Indique también la finalidad de la beca (para realizar la tesis, vinculada a un proyecto u otra finalidad) y si entre sus tareas de becario estaba la docencia.

Códigos	Organismo pagador
1	Ministerio de Educación y Ciencia
2	Otros Ministerios
3	Comunidad Autónoma
4	Centro Público de Investigación
5	Universidad
6	Institución o Fundación privada
7	Organismo internacional

Orden de beca	Organismo pagador (inserte código)	Finalidad (rodee con un círculo)		Docencia (rodee con un círculo)		Duración (en años y meses)
Primera beca		Tesis	1	Sí	1	
		Proyecto	2	No	2	
		Otra	3			
Segunda beca		Tesis	1	Sí	1	
		Proyecto	2	No	2	
		Otra	3			
Tercera beca		Tesis	1	Sí	1	
		Proyecto	2	No	2	
		Otra	3			
Cuarta beca		Tesis	1	Sí	1	
		Proyecto	2	No	2	
		Otra	3			

PASE A LA PREGUNTA 37

37. ¿Realizó durante el período predoctoral alguna estancia breve (entre 1 y 6 meses) en algún otro centro distinto a aquel al que estaba formalmente vinculado?

Sí	1	➔ Pase a la pregunta 38
No	2	➔ Pase a la pregunta 39

SI REALIZÓ ALGUNA ESTANCIA TEMPORAL EN LA ETAPA PRE-DOCTORAL
(«Sí» en la pregunta 37)

38. Por favor, señale dónde (centro, localidad y país), año y la duración aproximada en meses.

**Centro** (Universidad / Institución)	**Localidad y país**	**Fecha** (año)	**Duración** (en meses)

PASE A LA PREGUNTA 39

39. ¿Realizó publicaciones con su director de tesis durante la etapa predoctoral?

Sí	1
No	2

40. Durante la etapa predoctoral, ¿perteneció de forma continuada a algún grupo de investigación?

Sí	1
No	2

6 PUBLICACIONES Y MÉRITOS CIENTÍFICOS

Por último desearíamos conocer algunos datos referidos a sus *publicaciones* y a *otros méritos de carácter científico.*

41. **Por favor, le pedimos que haga un recorrido por su producción científica. Señale si cuenta o no con los siguientes méritos** (Por favor, rodee con un círculo en cada caso)

	Sí	No
Publicaciones en revistas del SCI, SSCI o AHCI *	1	2
Ponencias en congresos internacionales	1	2
Patentes nacionales registradas	1	2
Patentes europeas (EPO) registradas	1	2
Patentes en Estados Unidos registradas	1	2
Investigador principal (IP) de proyectos de I+D financiados con convocatorias públicas por *organismos españoles*	1	2
Participación en proyectos de I+D financiados con convocatorias públicas por *organismos españoles*	1	2
Investigador principal (IP) de proyectos de I+D financiados con convocatorias públicas por *organismos internacionales*	1	2
Participación en proyectos de I+D financiados con convocatorias públicas por *organismos internacionales*	1	2
Participación en contratos de I+D con empresas	1	2
Tesis dirigidas finalizadas	1	2
Miembro del Consejo de redacción o editor de una revista del SCI, SSCI o AHCI *	1	2
Realización de trabajos de consultoría	1	2

* Clave: SCI (Science Citation Index); SSCI (Social Science Citation Index); AHCI (Arts and Humanities Citation Index)

42. Para finalizar, señale el nombre de las revistas científicas en las que hayan aparecido los tres artículos que usted considere más importantes dentro de su carrera, así como su año de publicación.

Nombre de la revista	Año de publicación

Si hay algo que desea añadir acerca de la carrera académica e investigadora o cualquier otro comentario sobre los temas tratados en este cuestionario, por favor hágalo a continuación:

El resumen de los resultados de este estudio estará disponible en la página web:

www.iesam.csic.es/spritte.htm

También si lo desea, puede indicarnos el correo al que quiere que le enviemos el resumen:

Por favor introduzca el cuestionario cumplimentado en el sobre franqueado que le acompaña.

MUCHAS GRACIAS POR SU COLABORACIÓN.

MINISTERIO
DE EDUCACIÓN
Y CIENCIA
