# Supplementary material for: Time to Tenure in Spanish Universities: An Event History Analysis
Source: PLoS One. 2013 Oct 8;8(10):e77028. doi: 10.1371/journal.pone.0077028 (PMC3792917; doi:10.1371/journal.pone.0077028)
Supplement: File S3 — Annex: Model specification. (DOC) [file pone.0077028.s003.doc]

**File S3. Annex. Log-logistic model specification**

The log-logistic model is the optimal specification for the transition from PhD to a tenure position (see Section 4). The log-logistic model has two parameters: the scale parameter $a$ and shape parameter $b$. The **transition rate** model has the form:

$r(t)=\frac{ba^{b}t^{b-1}}{1+\left(at\right)^{b}}$,

where the rate is monotonically decreasing if $b\leq1$, and the rate of obtaining tenure decreases over the years; while if it is greater than one, the transition rate is bell shaped (inverted U), and the rate increases to a maximum and then decreases. The scale parameter and the shape parameter are expressed as:

$a=\exp\{-(\alpha_{0}+\alpha_{1}X_{1}+\alpha_{2}X_{2}...+\alpha_{p}X_{p})\}$ (1)

and $b=\exp\{-\beta_{0}\}$, (2)

where $\alpha_{1},\alpha_{2},...,\alpha_{p}$ are the model coefficients, $p$ is the number of coefficients, and $\beta_{0}$is the coefficient */ln_gam* of the report Stata.[[1]](#footnote-2)

The **density function**, **survival**, and **expected duration** of the log-logistic model are defined:

$$f(t)=\frac{ba^{b}t^{b-1}}{\left(1+\left(at\right)^{b}\right)^{2}}$$

$S(t)=\frac{1}{1+\left(at\right)^{b}}$ (3)

$E(T)=\frac{1}{a}\times\frac{\pi}{b\sin\left(\frac{\pi}{b}\right)}$ (4)

Considering the value of */ln_gam* = -**1.30** (Table 6, main text) we calculate from equation (2)

$$b=\exp\{-(-\text{1.30)}\}=3.69$$

1. The analysis was conducted using STREG in STATA. [↑](#footnote-ref-2)
